# Exploring PAZ/3′-overhang interaction to improve siRNA specificity. A combined experimental and modeling study†
†Electronic supplementary information (ESI) available. See DOI: 10.1039/c8sc00010g


**DOI:** 10.1039/c8sc00010g

**Published:** 2018-01-15

**Authors:** Adele Alagia, Andreia F. Jorge, Anna Aviñó, Tânia F. G. G. Cova, Ramon Crehuet, Santiago Grijalvo, Alberto A. C. C. Pais, Ramon Eritja

**Affiliations:** a Institute for Advanced Chemistry of Catalonia (IQAC-CSIC) , Jordi Girona 18-26 , E-08034 Barcelona , Spain . Email: recgma@cid.csic.es ; Email: adele.alagia@gmail.com ; Tel: +34 934006145; b Networking Center on Bioengineering, Biomaterials and Nanomedicine (CIBER-BBN) , Jordi Girona 18-26 , E-08034 Barcelona , Spain; c CQC , Department of Chemistry , University of Coimbra , Rua Larga , 3004-535 Coimbra , Portugal . Email: andreiaj@qui.uc.pt

## Abstract

The understanding of the dynamical and mechanistic aspects that lie behind siRNA-based gene regulation is a requisite to boost the performance of siRNA therapeutics. A systematic experimental and computational study on the 3′-overhang structural requirements for the design of more specific and potent siRNA molecules was carried out using nucleotide analogues differing in structural parameters, such as sugar constraint, lack of nucleobase, distance between the phosphodiester backbone and nucleobase, enantioselectivity, and steric hindrance. The results established a set of rules governing the siRNA-mediated silencing, indicating that the thermodynamic stability of the 5′-end is a crucial determinant for antisense-mediated silencing but is not sufficient to avoid sense-mediated silencing. Both theoretical and experimental approaches consistently evidence the existence of a direct connection between the PAZ/3′-overhang binding affinity and siRNA’s potency and specificity. An overall description of the systems is thus achieved by atomistic simulations and free energy calculations that allow us to propose a robust and self-contained procedure for studying the factors implied in PAZ/3′-overhang siRNA interactions. A higher RNAi activity is associated with a moderate-to-strong PAZ/3′-overhang binding. Contrarily, lower binding energies compromise siRNA potency, increase specificity, and favor siRNA downregulation by Ago2-independent mechanisms. This work provides in-depth details for the design of powerful and safe synthetic nucleotide analogues for substitution at the 3′-overhang, enabling some of the intrinsic siRNA disadvantages to be overcome.

## Abstract 2

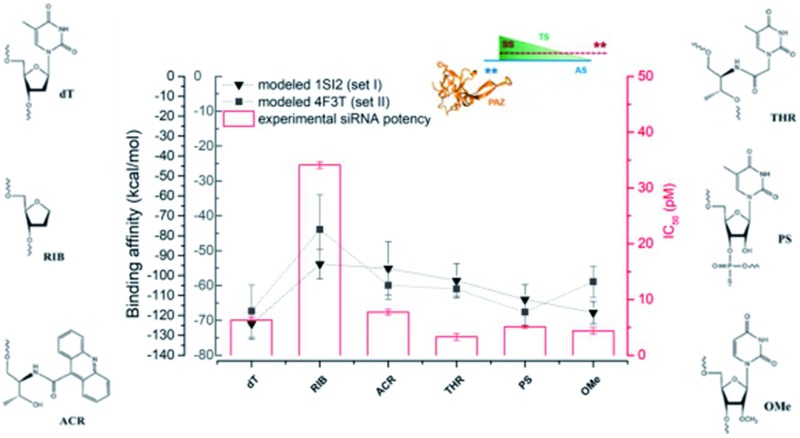
A direct connection between the PAZ/3′-overhang binding affinity and the siRNA potency and specificity is defined through complementary experimental and computational results.

## Introduction

The discovery of the RNA interference (RNAi) pathway^1^ and the identification of the small interfering RNA (siRNA) molecule as an RNAi trigger^2^ have simplified the study of gene function and attracted a great deal of attention to the development of next-generation medicine, able to treat any disease-related gene.^3^,^4^ Over the past 15 years, remarkable advances were reported in the development of siRNA-based therapeutics, enlarging the number of ongoing clinical trials and patents in this field.^5^,^6^ The challenges in converting siRNA molecules into efficient drugs are their easy degradation under physiological conditions, their unwanted gene silencing, commonly called off-target effects, their potential to trigger innate immune responses, and their low ability to be internalized in cellular compartments. To circumvent these intrinsic siRNA disadvantages, two strategies are commonly adopted: (i) the chemical modification of the basic building blocks of the natural siRNA and (ii) the incorporation of siRNA molecules into smart vehicles to facilitate cell delivery.^6^,^7^ The development of chemical modifications has been the focus of recent research, in which attention has been directed towards RNA modifications that enable the avoidance of sense strand-mediated and miRNA-like off-targeting effects.^8^–^12^

In more detail, the ability of the RNAi machinery to control gene expression essentially relies on preventing translational initiation,^13^ while in contrast, the introduction of synthetic siRNA molecules into cells results in sequence-specific endonucleolytic cleavage of the target mRNA.^14^ The siRNA molecules are short double-stranded RNAs, approximately 21 nucleotides (nt) in length, bearing 2-nt overhangs at both 3′-ends. One strand is named antisense (AS) or guide, whereas the complementary strand is termed sense (SS) or passenger. The siRNA molecule is initially incorporated into the RISC in double stranded fashion, then a process called RISC maturation results in the dissociation of siRNA SS from AS, permitting the recognition of the target messenger RNA (mRNA).^15^ The process of siRNA loading requires the action of the RISC-Loading Complex (RLC), which includes the DICER, Argonaute2 (Ago2) and TRBP (HIV-1 TAR RNA Binding Protein) proteins.^16^,^17^ The Ago2 protein, known as the slicing effector of the RNAi process, is a cradle-shaped protein of four domains: MID (middle), PIWI (P-element-induced wimpy testes), PAZ (Piwi/Argonaute/Zwille), and N-terminal. The PIWI domain folds into an RNase H-like structure and contains the catalytic triad “DDH” necessary for slicing activity. The N-terminal domain, acting as a molecular wedge, supports the siRNA unwinding process during the RISC maturation step. Both termini of the siRNA AS bind to the MID and PAZ domains; the MID anchors the 5′-phosphorylated end whereas the 3′-overhang is buried into the PAZ cleft.^16^,^18^–^20^ The incorrect loading of the siRNA strand into RISC has double negative effects (i) the downregulation of those RNA sequences complementary to SS and (ii) the reduction of the freely available RISC for AS-mediated silencing.^21^ The synthetic siRNA may in addition function like a natural microRNA (miRNA), suppressing the translation of genes through the interaction between the seed region of siRNA AS, in positions 2–8 from 5′-end, with the 3′-UTR of mRNA only requiring partial homology.^21^

The combination of computational and experimental methodologies has recently permitted description of the Ago2 conformational changes (*e.g.* the two-state model of the PAZ/3′-overhang assembly and the lodge/dislodge motion of the AS 3′-overhang from the PAZ domain) essential for siRNA unwinding, target recognition and slicer-dependent silencing.^22^,^23^ To achieve valuable insights about the role of the Ago2’s PAZ domain in gene silencing, diverse theoretical approaches, such as protein-siRNA docking,^24^–^26^ all atom molecular dynamics (MD) simulations^27^,^28^ or the combination of more than one approach, have been employed.^23^,^29^–^31^ The introduction of natural and chemical modifications at the 3′-overhang,^24^,^26^–^28^ 5′-end,^25^,^32^,^33^ central positions and seed region^29^,^34^ of the siRNA molecule has allowed for understanding their effect on the Ago2 functional mechanisms. In addition, the siRNA properties, such as thermodynamic asymmetry, A-form helix, central mismatches/bulges, and target strand accessibility have been determined as pivotal hallmarks for siRNA-mediated gene silencing.^35^–^38^ A direct correlation between the PAZ/3′-overhang binding affinity and siRNA potency/specificity has been suggested through experimental and theoretical approaches.^34^,^39^,^40^ Nevertheless, there is still controversy about whether the presence of a PAZ domain is important for siRNA recognition, and also, whether stronger or weaker binding with the PAZ domain facilitates or hampers RNAi activity.^24^,^30^,^41^–^43^ Despite recent advances, a systematic study on how the PAZ/3′-overhang binding affinities are modulated by the 3′-overhang structure is still lacking. Also, no systematic studies on SS-mediated off-target silencing have been reported.

**Fig. 1:**
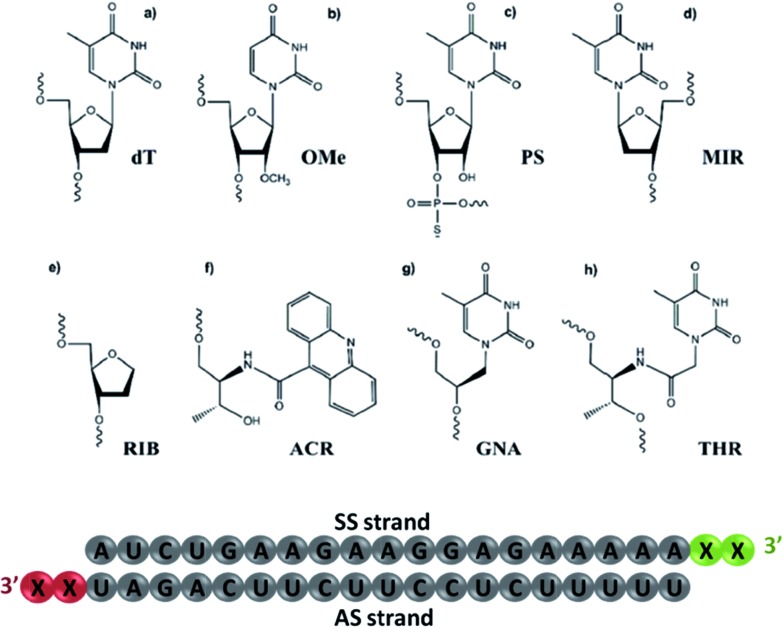
The chemical modifications used in this study. (a) 2′-Deoxythymidine (dT); (b) 2′-*O*-methyl-uridine (OMe); (c) phosphorothioate thymidine (PS); (d) β-l-2′-deoxythymidine (MIR); (e) 1,4-anhydro-2-deoxy-d-ribitol (RIB); (f) acridinyl-l-threoninol (ACR); (g) thymine glycol nucleic acid (GNA) and (h) l-threoninol-thymine (THR). The schematic representation of siRNA illustrates the nucleotide sequence, where X defines the position for chemical modification conjugation.

Herein, we aim to analyze experimentally the contribution of the 3′-overhang structure in the AS-/SS-mediated silencing of siRNA molecules bearing chemical modifications at the 3′-overhang, including thymidine (dT), 2′-*O*-methyl-uridine (OMe), phosphorothioate thymidine (PS), l-threoninol-acridine (ACR), 2-deoxyribitol (RIB), thymine glycol nucleic acid (GNA), β-l-thymidine (MIR) and l-threoninol-thymine (THR) (see Fig. 1). The importance of both siRNA overhangs is also assessed using siRNA with blunt ends. Further insights into the potential role of TRBP and Ago2 in AS-/SS-mediated silencing and cleavage-dependent gene downregulation are also provided. In a complementary part, a complete theoretical study based on MD simulations is performed to evaluate the thermodynamic and structural parameters involved in the interaction of the most representative analogs studied experimentally with the PAZ domain of human Argonaute (hAgo).

## Results

### Silencing potencies of 3′-modified siRNAs

To investigate the contribution of the 3′-overhang to siRNA potency, we first modified the end nucleotides of the AS- and SS-siRNA strands, see Fig. 1 and Tables S1 and S2† for the siRNA design details. The 3′-overhang modifications can be generally sorted into five classes: (i) thymidine (dT), 2′-*O*-methyl-uridine (OMe), and phosphorothioate (PS), belonging to commonly used residues; (ii) thymine glycol nucleic acid (GNA) and l-threoninol-thymine (THR), two acyclic nucleotide mimetics; (iii) β-l-thymidine (MIR), an enantiomer of the natural thymidine; (iv) l-threoninol-acridine, an acyclic derivative attached to a bulky aromatic ring system (ACR), and (v) 2-deoxyribitol (RIB), an abasic derivative. These modifications were selected to provide complete insight into the role of the physico-chemical nature of modified nucleotides on the siRNA potency, such as sugar constraint, lack of nucleobase, distance between the phosphodiester backbone and nucleobase, enantioselectivity, and steric hindrance.

As a measure of the siRNA potency, the half-maximal inhibitory concentration (IC_50_) values were quantified in the HeLa cells using dual luciferase assay (see Table 1). siRNA modified with two dT nucleotides at the 3′-overhang (wt) was considered as the control. As shown in Table 1, the lack of nucleobase at the AS 3′-overhang (RIB2) has a detrimental effect on the siRNA efficiency causing a drastic reduction in the IC_50_ values to about 5-fold lower with respect to the wt (RIB2 = 34.1 pM *vs.* wt = 6.3 pM). A reduction in the siRNA potency was also observed for the siRNA carrying MIR (MIR2 = 8.2 pM) and ACR (ACR2 = 7.7 pM) demonstrating that there might be some degree of enantioselectivity in PAZ recognition and confirming the negative effect of the bulky aromatic derivatives at the 3′-end of the AS, as described elsewhere.^38^,^44^ The GNA and PS moieties gave rise to similar IC_50_ values, GNA2 = 6.4 pM and PS2 = 5.1 pM, resembling the value obtained for wt. OMe and THR are clearly the most active modifications applied (OMe2 = 4.4 pM and THR2 = 3.3 pM), but the conjugation of THR in the 3′-overhang of AS leads to the highest degree of siRNA silencing.

**Table 1:** The half maximal inhibitory concentration (IC_50_, pM) of 3′overhang-modified siRNAs

siRNA	3′-End, AS strand	3′-End, SS strand	Potency (IC_50_ ± SD)
wt	dT	dT	6.3 ± 0.5
OMe2	OMe	dT	4.4 ± 0.6
PS2	PS	dT	5.1 ± 0.3
RIB2	RIB	dT	34.1 ± 0.5
ACR2	ACR	dT	7.7 ± 0.5
MIR2	MIR	dT	8.2 ± 0.7
GNA2	GNA	dT	6.4 ± 0.4
THR2	THR	dT	3.3 ± 0.6
OMe3	dT	OMe	5.6 ± 0.5
PS3	dT	PS	7.3 ± 0.2
RIB3	dT	RIB	8.2 ± 0.4
ACR3	dT	ACR	4.3 ± 0.7
MIR3	dT	MIR	9.1 ± 0.4
GNA3	dT	GNA	5.9 ± 0.5
THR3	dT	THR	7.5 ± 0.4

Theoretically, due to the rational design of our siRNA sequence,^4^,^38^ the modification of AS 3′-overhang with high-affinity derivatives for PAZ binding should enhance the preferential incorporation of the AS leading to weaker SS-mediated silencing. The values of IC_50_ obtained for siRNAs modified at the SS present a narrow distribution, showing a lower contribution of the complementary strand to the overall potency of siRNA. In fact, the potency of siRNA is considered to be interconnected with the binding affinity of the PAZ/3′-overhang siRNA, according to the two-state model of Argonaute action.^22^,^23^ A rationale for these results will be provided by MD simulations.

### The 3′-overhang structural features of the canonical siRNA duplex affect the SS-mediated off-target silencing

For the success of siRNA therapeutics, the development of chemical modifications able to attain, simultaneously, high silencing efficiency and on-target specificity is required to overcome the inherent siRNA hindrances. In order to evaluate the influence of the chemical modifications anchored at the 3′ overhang, either in the AS- or SS-mediated silencing, psiCHECK2 vectors assay was carried out.

**Fig. 2:**
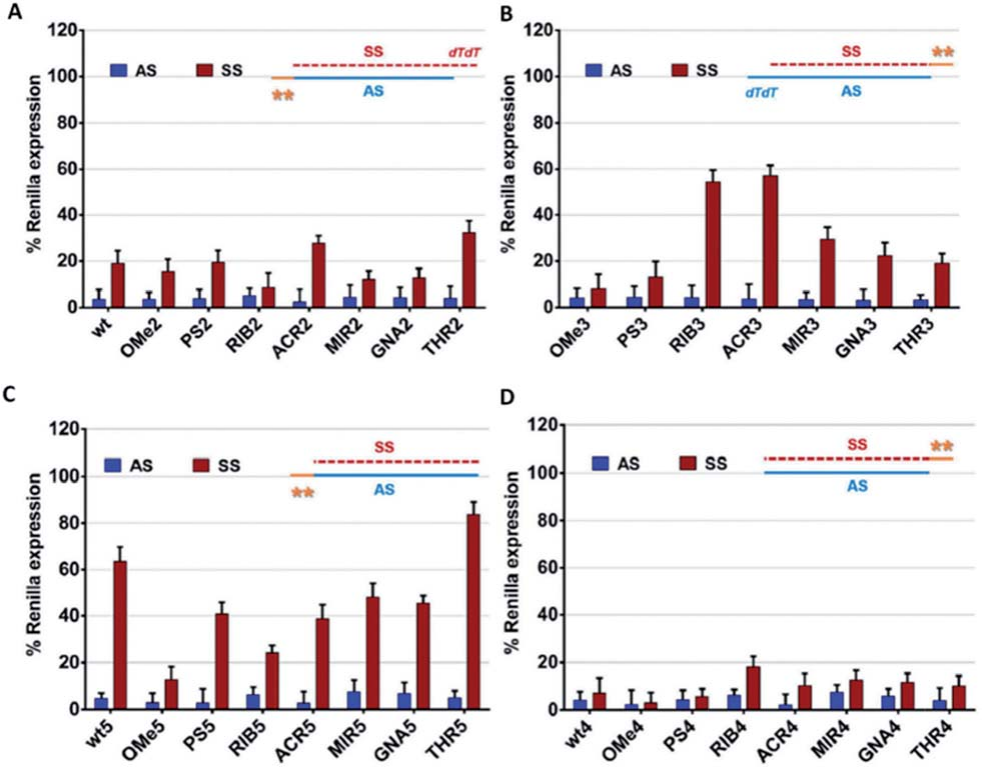
The AS/SS-mediated silencing analysis of the canonical siRNA molecules bearing chemical modifications at the 3′-overhang. The knockdown efficiency of the antisense (AS) and sense (SS) strands is plotted as a percentage (SD; *n* = 3) of the normalized *Renilla* luciferase expression. The luciferase activity of the mock transfected cells (only vectors) was designed as 100%. All tested siRNAs were transfected at a concentration of 1 nM and the silencing activities were measured at 24 h post-transfection. (A) unmodified (wt) and AS 3′-overhang modified siRNAs (OMe2, PS2, RIB2, ACR2, MIR2, GNA2, and THR2), (B) SS 3′-overhang modified siRNAs (OMe3, PS3, RIB3, ACR3, MIR3, GNA3, and THR3), (C) unmodified (wt5) and AS 3′-overhang modified siRNAs (OMe5, PS5, RIB5, ACR5, MIR5, GNA5, and THR5) and blunt-ended SS, and (D) unmodified (wt4) and SS 3′-overhang modified siRNAs (OMe5, PS5, RIB5, ACR5, MIR5, GNA5, and THR5) and blunt-ended AS.

Comparing the data shown in Fig. 2A and B, a high consistency between the values is obtained for AS-mediated silencing, revealing in all cases high silencing efficacy. This suggests that siRNA specificity is mainly driven by the thermodynamic stability of the siRNA duplex and secondarily affected by the 3′-overhang composition. SS-mediated silencing is generally less efficient in *Renilla* knockdown, and interestingly, this response is strongly influenced by the composition of the 3′-overhang. For instance, the introduction of RIB (RIB3) and ACR (ACR3) moieties at the SS 3′-overhang produces a reduction of the SS-mediated silencing, enhancing the siRNA silencing specificity (Fig. 2B). In the presence of OMe3 and PS3, the values of AS- and SS-mediated silencing are comparable. A fairly degree of silencing specificity is found for SS modified with MIR3, GNA3 and THR3. Interestingly, the modification of AS with THR, correspondent to THR2, promotes the highest silencing potency (Table 1), and in addition, its level of silencing specificity is also the highest among all modifications tested (Fig. 2A).

Our data demonstrates that through chemical modification of the 3′ overhangs of both AS and SS, we can modulate the extent of SS-mediated off-target silencing, proving not only the importance of this overhang to RISC incorporation but also that this approach is valuable for improving siRNA-driven specificity.

### The presence and nature of the 3′-overhang regulate the SS-mediated off-target silencing

To further examine the correlation between the modification at the 3′-overhang and siRNA silencing specificity, the 3′-overhang was trimmed from both the siRNA strands and the AS-/SS-mediated silencing was measured, see Fig. 2C and D.

Comparing the references of the blunt-ended siRNAs, wt4 and wt5, with the general reference, wt (Fig. 2A), differences in the *Renilla* expression can be seen, especially when the SS overhang is trimmed. Indeed, looking at the AS/SS silencing profile of the SS-blunt ended siRNAs (Fig. 2C), a general improvement of the siRNA specificity is observed, compared to the canonical siRNAs (Fig. 2A), confirming that the design of structurally asymmetric siRNAs could be exploited for the production of siRNA molecules with a higher degree of specificity.

An exception is found for OMe modification (OMe5), where the results with blunt-ended SS mirror the AS/SS silencing abilities of the canonical siRNAs OMe2 and OMe3. A large improvement of the siRNA specificity is obtained by the modification of the AS 3′-overhang with THR residues with blunt-ended SS, eliminating almost completely the SS-mediated off-target silencing. In addition, data illustrates that the impairment of SS-mediated silencing depends on the nature of the AS 3′-overhang, and a good correlation exists between the data of Fig. 2A and C, despite the distinct magnitudes.

On the other hand, if the 3′-overhang belonging to AS is trimmed, regardless of the modification in the SS 3′-overhang, the SS-mediated silencing of the blunt-ended antisense siRNAs (Fig. 2B) is powerful, and the siRNA specificity is poor. These findings provided further evidence on the hypothesis claiming a rule hierarchy for strand incorporation and SS-mediated silencing. The strong activity of AS (≥95% of *Renilla* expression) leads to consideration of the siRNA thermodynamic asymmetry as the principal determinant for siRNA activity, while the composition and presence of the siRNA 3′-overhangs, acting as auxiliary elements, are especially important for siRNA specificity.

### The AS-mediated silencing profile of the AS 3′-overhang modified siRNAs depends on the cellular competitive environment

**Fig. 3:**
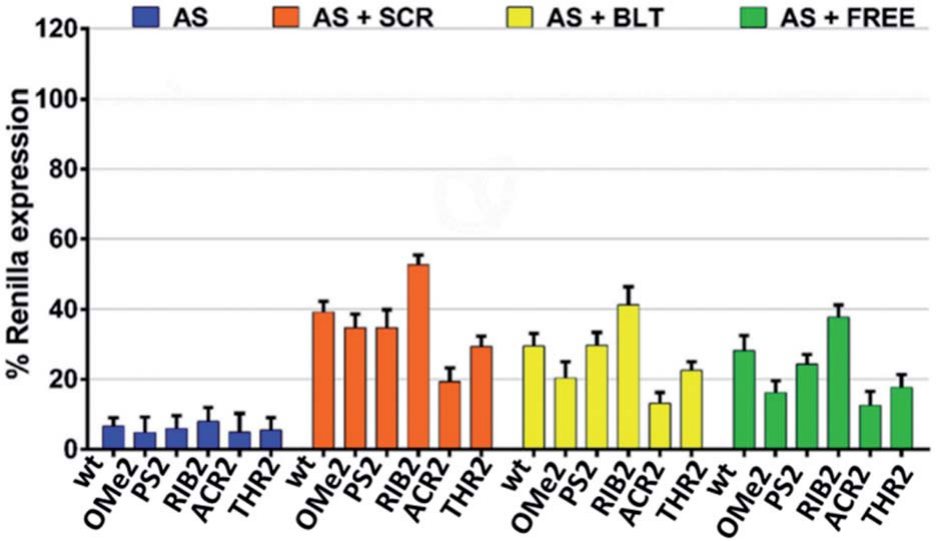
The AS-mediated silencing of the antisense 3′-overhang modified siRNA molecules in the presence of non-active siRNA competitors; SCR (scrambled non-active siRNA bearing both 3′-overhangs), BLT (scrambled non-active siRNA without both 3′-overhangs) and FREE (non-active RISC-free siRNA). siRNA competitors were added 4 h prior to siRNA transfection at a concentration of 30 nM. The AS 3′-overhang modified siRNA molecules were transfected at a concentration of 1 nM and the silencing activities were measured at 24 h post-transfection (*n* = 3 ± SD). As a control, the luciferase activity of the cells transfected with the vector alone and vector plus competitors were assessed. No downregulation of the *Renilla* expression was observed. The luciferase activity of mock transfected cells (vector plus competitors for AS + SCR, AS + BLT, AS + FREE, or the vector alone for AS) was set as 100%.

Next, we sought to evaluate how the silencing profile of our modified siRNA molecules might change in the presence of non-active siRNAs with different competitive characteristics. In what follows, we focused our attention on the modifications that were shown to be more relevant in terms of their silencing potency and specificity. We reasoned that the competition between an active and non-active (scramble) siRNA bearing both 3′-overhangs might impact stronger on the siRNA activity compared to a competitor lacking both 3′ dangling ends. In light of this hypothesis, we assessed the AS-mediated silencing of the siRNAs bearing the dT, OMe, PS, RIB, ACR, and THR modifications in the presence of canonical scrambled siRNA (SCR), blunt-ended scrambled siRNA (BLT), as well as free-RISC siRNA (unable to be loaded into the Ago proteins) (Fig. 3).

As expected, the competition is more pronounced in the presence of scramble siRNA bearing both 3′-overhangs (AS + SCR) compared to the blunt-ended competitor (AS + BLT). The trend obtained for modified siRNA is reproducible for all competitors tested, showing an AS-mediated silencing activity of the wt, OMe2, PS2, and THR2 siRNAs stronger than for RIB2 and ACR2 siRNA. Interestingly, ACR2 siRNA is always the most effective in silencing and maintains approximately the same percentage of silencing in the presence of any competitor.

These data indicate that the incorporation of siRNA with overhangs is preferred over the incorporation of their structurally blunt-ended variants, thereby corroborating the AS- and SS-mediating findings. Furthermore, the different behavior found for ACR might suggest additional non-specific interactions that should be explored in more detail.

### The presence of the TRBP protein plays a role in AS/SS-mediated silencing

**Fig. 4:**
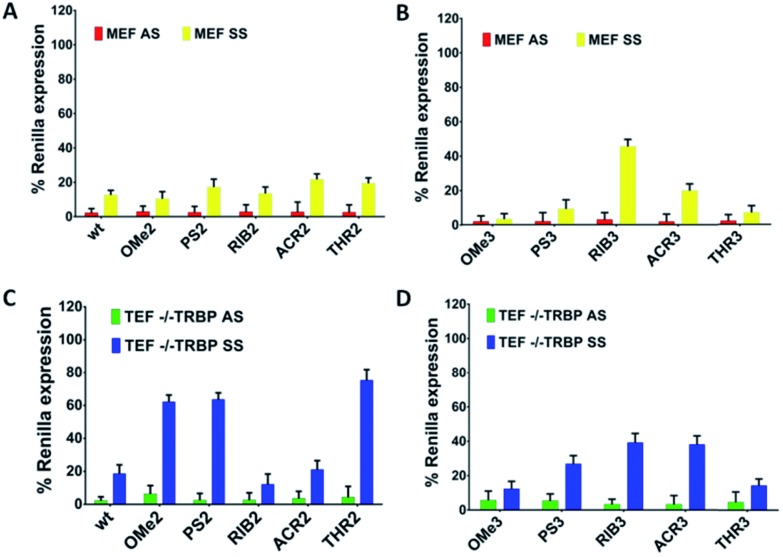
The AS/SS-driven silencing profile of the canonical siRNA molecules in the MEF and TEF TRBP^–/–^ cells. The knockdown efficiency of the antisense (AS) and sense (SS) strands is plotted as a percentage (*n* = 3 ± SD) of the normalized *Renilla* luciferase expression. The luciferase activity of the mock transfected cells (only vectors) was designed as 100%. All the tested siRNAs were transfected at a concentration of 1 nM and the silencing activities were measured at 24 h post-transfection in the MEF and TEF TRBP^–/–^ cells.

The TRBP protein is an important RNAi co-factor involved in Dicer processing and in sensing the siRNA asymmetry.^17^ The Dicer/TRBP complex binds the siRNA directionally: siRNA’s less stable end is preferentially bound by Dicer, whereas TRBP interacts with the more stable end. In order to determine whether the TRBP asymmetry sensing features might be important for the siRNA specificity, we transfected the MEF and TEF TRBP^–/–^ cells with several modified siRNA molecules (Fig. 4A–D). In the presence of TRBP, the siRNA specificity showed similar silencing pattern in both the MEF and HeLa cells (Fig. 2 and 4), ACR3 being the only exception.

For SS-mediated silencing, the absence of TRBP affects the silencing to the largest extent. As a general trend, a reduction of the SS-mediated silencing is observed for the modifications of OMe, PS and THR conjugating to AS (OMe2, PS2, and THR2). Conversely, the siRNAs RIB2 and ACR2 preserve the percentages of silencing either in the presence or absence of TRBP. In the case of having the SSs modified, in general, a reduction can be seen in SS-mediated silencing for modifications OMe3, PS3, ACR3 and THR3 and there is no effect on RIB3. Thus, the selection carried out by TRBP seems to depend on the nature of the 3′-overhang modifications and on the strand selected.

These data suggest that, without the TRBP protein, the 3′-overhang structural asymmetry plays a stronger role in siRNA specificity, but interestingly, there are exceptions for RIB2 and ACR2. Likely, the absence of TRBP that bridges Dicer to Ago2 might preferentially commit the sensing of the siRNA asymmetry to the Ago2 protein.

### The structure of the 3′-overhang is decisive for Ago2-dependent gene silencing

**Fig. 5:**
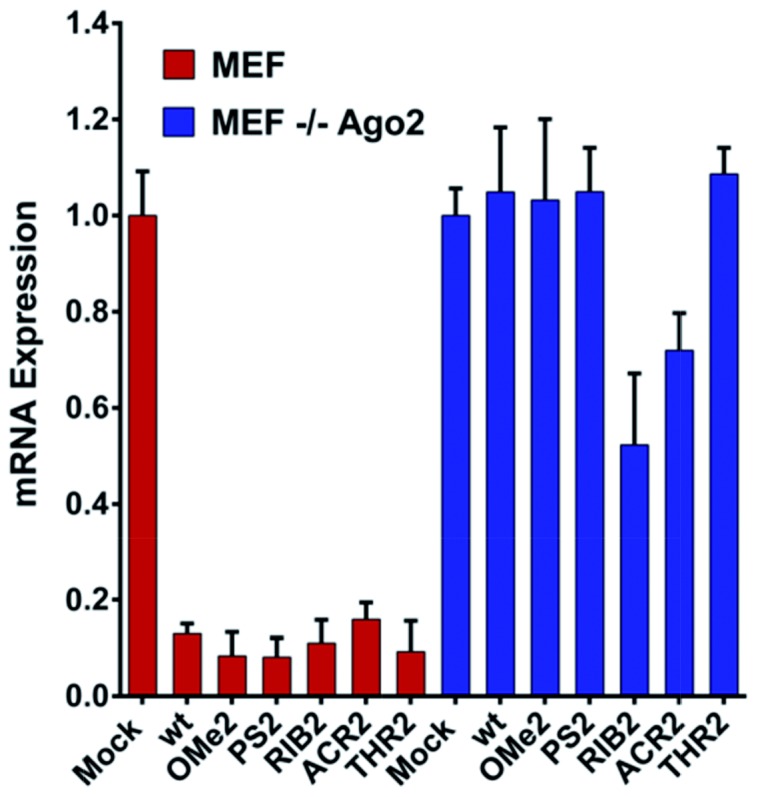
The siRNA silencing ability in the MEF and MEF Ago2^–/–^ cells. The *Renilla* mRNA reduction in the MEF and MEF Ago2^–/–^ cells. 1 nM of the unmodified (wt) and modified (OMe2, PS2, RIB2, ACR2, and THR2) siRNAs were co-transfected with a psiCHECK2 (AS) reporter. After 24 h, the cells were harvested for RNA extraction and qRT-PCR measurement [*n* = 3 ± SD]. The mock transfection, only the psicheck2 AS vector, was set as 1.

Upon observing large differences in the silencing potencies and specificities imposed by our modified siRNAs, along with some pieces of evidence supporting the existence of different pathways of silencing, we wondered how these modifications might impact on the Ago2 function. To investigate whether the improper recognition by Ago2 and/or altered hybridization with target mRNA is the basis of the silencing outcomes, the Ago2-mediated silencing was inspected (Fig. 5).

Interestingly, our results show that the RIB2 and ACR2 siRNAs are able to downregulate the target mRNA in the MEF and MEF Ago2^–/–^ cells. Indeed, RiB2 can effectively reduce mRNA silencing in *ca.* 50% in the absence of Ago2. In contrast, OMe2, PS2 and THR2 are shown to completely abolish their silencing effect, when transfected in cells without Ago2. The Ago2-independent silencing ability of ACR2 and RIB2 might rely on Ago1-mediated silencing or enhanced mRNA decay. Thereby, these results clearly demonstrate that, depending on the modifications introduced at the 3′-overhang of siRNA, different siRNA silencing pathways are activated.

### The interaction between the modified siRNA 3′-overhangs and the PAZ domain is modeled by molecular dynamics

**Fig. 6:**
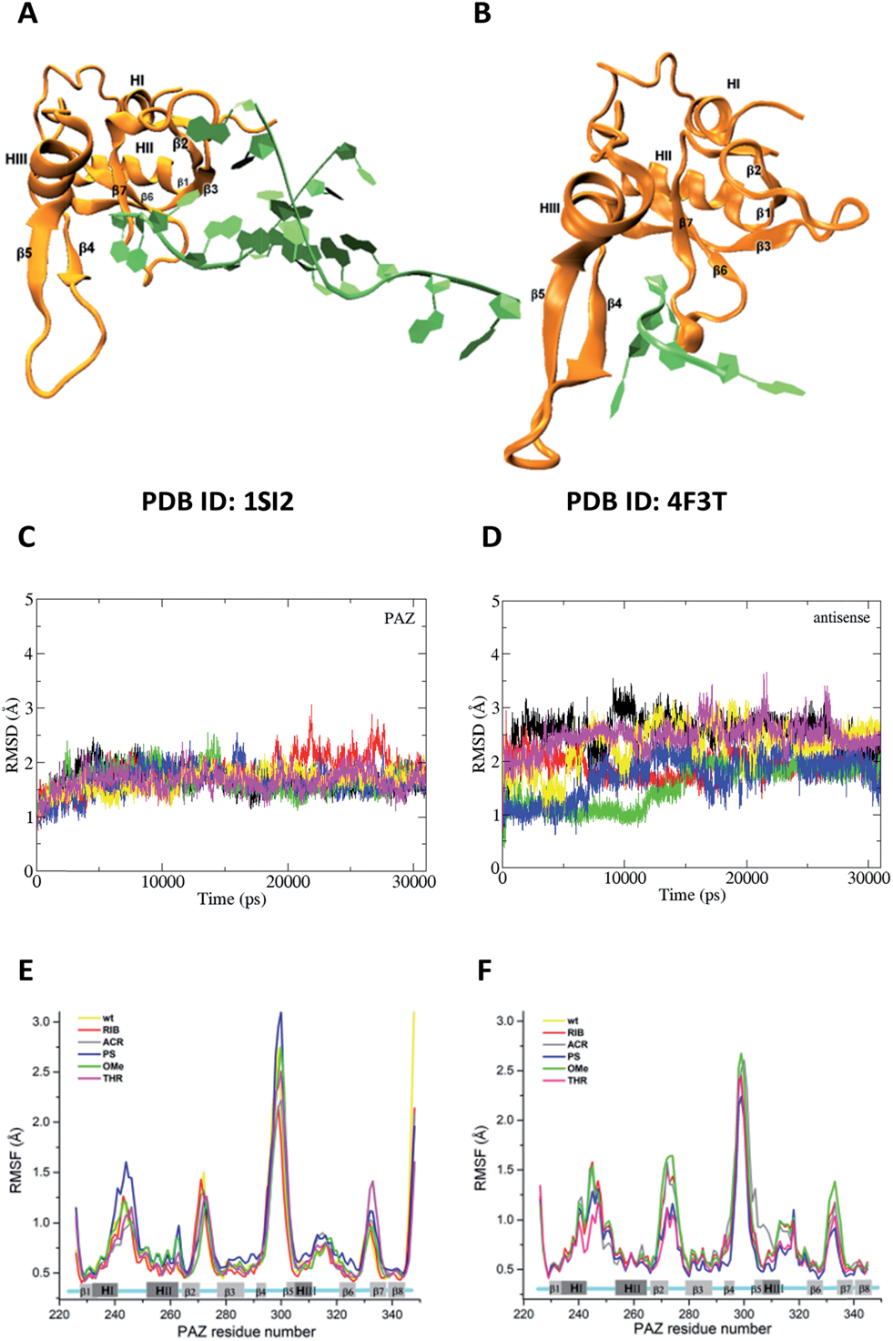
The feasibility and structural stability of the MD simulations. (A) and (B) represent the crystal structures used as the initial atomic coordinates for MD simulations after addition and minimization of the missing amino acid residues. In (A), the structure of PAZ from hAgo1 bound to the double-stranded 9-mer siRNA was taken from PDB ID 1SI2. In (B), the PAZ domain from hAgo2 bound to 4-mer miRNA was obtained from PDB ID ; 4F3T. The RNA strands are colored in green whereas the PAZ domains are colored in orange. The 3′-overhang of siRNA is bound to the PAZ cleft generated by α-helix 3 (HIII) and β-sheet 4 (β4) along one face and the β7 and the loop produced between along the opposite face. (C) and (D) are the RMSD profiles for PAZ and siRNA-dT–dT3′ corresponding to set II, calculated from the heavy atoms with the C_α_ atoms pertaining to the PAZ backbone and from RNA heavy atoms (P, O3′, O5′, C3′, C4′, and C5′). The first frame of production simulation was used as the RMSD reference structure. Each RMSD curve corresponds to the 6 independent MD replicas. (E) and (F) show the RMSF for the backbone atoms of the PAZ domain from the hAgo1 and hAgo2 bound to the unmodified and modified siRNAs, respectively. Each RMSF curve represents the average of the analyses from the 6 MD simulations.

The experimental findings consistently indicate a dependency of the measured parameters on the nature of the chemical modifications introduced at the last two nucleotides of the 3′-overhang. During the mechanism of silencing, the 3′-region of the siRNA binds, releases and rebinds to the PAZ domain in a cyclic manner, making this dynamic interaction reportedly essential for RNAi activity.^45^,^46^ Here, hypothesizing that the PAZ/3′-overhang interaction plays a role in the trends reported experimentally, we carried out MD simulations to quantitatively evaluate the ability of each modified compound to bind PAZ domain. All the siRNA molecules modeled are equivalent to those studied experimentally in terms of the 3′-overhang modifications and nucleotide sequence. The simulated systems are summarized in Table 2. We compared the energetic data obtained from the simulation of the two distinct X-ray crystal coordinates for PAZ/3′-overhang RNA (PDB ID: ; 1SI2 (set I)^47^ and PDB ID: ; 4F3T (set II))^18^ see Fig. 6A and B.

**Table 2:** Summary of the PAZ/siRNA complexes under study

Set/PDB code	Simulated structure	Simulation length (ns)	Number of replicates
Set I/1SI2	PAZ: RNA dT–dT3′	31	6
	PAZ: RNA RIB–RIB3′	31	6
	PAZ: RNA ACR–ACR3′	31	6
	PAZ: RNA PS–PS3′	31	6
	PAZ: RNA OMe–OMe3′	31	6
	PAZ: RNA THR–THR3′	31	6
Set II/4F3T	PAZ: RNA dT–dT3′	31	6
	PAZ: RNA RIB–RIB3′	31	6
	PAZ: RNA ACR–ACR3′	31	6
	PAZ: RNA PS–PS3′	31	6
	PAZ: RNA OMe–OMe3′	31	6
	PAZ: RNA THR–THR3′	31	6

To cover a broader conformational space, we decided to start sampling from these two alternative crystal structures and perform a set of independent short simulations for all the modified siRNAs, each starting with different initial velocities. It is known that Ago1 and Ago2 share 82% of their amino acid sequence.^48^ In this section we will assess whether the differences between the proteins affect their interaction with siRNA. Eventually, if differences occur in the interaction of the substituted siRNA and PAZ domain from the distinct Ago families, they might provide some insight on the pathway followed by RIB and ACR modified siRNAs. The details of the simulation procedures are provided in the Materials and methods section of the ESI.†

For all simulated systems, the conformational fluctuations of the PAZ and RNA structures were inspected in terms of the root-mean-square displacement (RMSD) with respect to the starting structures (Fig. 6, S1 (set I) and S2 (set II)†). In set II, both PAZ and 4-nt RNA dT–dT3′ were found to be stable along the production run (Fig. 6C and D), and also for other modified siRNAs (Fig. S2†). A notable exception was detected for the PAZ domain in the presence of siRNA modified with ACR (Fig. S2C†), where a higher fluctuation of the RMSD values is observed. In set I, the lower fluctuation of the RMSD values indicates a stable behavior of PAZ and the RNA AS, whereas a higher drift was detected for SS (Fig. S1†). This may be related to a poor SS/PAZ contact and a low base pairing with AS (with only 7-bp matches). In general, the use of independent simulations allowed us to explore a large conformational space, as shown by the distinct RMSD ranges attained individually. The low stability observed in some replicas can be ascribed to the starting structures, which were randomly generated by computing different initial velocities to produce independent runs. In all cases, stable values of the RMSD were observed in 31 ns, indicating a sufficiently long simulation time.

The dynamic character of the PAZ domain is supported by tracking the C_α_ atoms of the PAZ backbone for all systems, using standard root-mean-square fluctuation (RMSF) analyses (Fig. 6E and F). In the literature, the PAZ domain is reported as the most flexible domain of Ago proteins.^30^,^31^ Comparing with the unmodified system, siRNA with 3′-overhang bearing dT, similar RMSF trends are observed in the modified siRNA systems, with deviations associated only with the flexible loops. For set I, the destabilization of the loops comprised between α-helix-I and II (HI–HII) and β-strands 4 and 5 (β4–β5) is higher in the presence of RNA modified with PS, albeit quite similar to the ones modified with OMe, dT and THR. In contrast, ACR and RIB tend to stabilize the region comprised between β4–β5. For set II, the substitution with PS promotes a reduction in the PAZ residue fluctuation in almost all flexible loops. siRNA-THR–THR3′ follows exactly the same trend as siRNA modified with PS. The ACR modification induces a destabilization of the residues located in the loop formed between β4–β5 and α-helix 3 (HIII).

### The modification in the 3′-overhang affects the affinity of binding in the PAZ/siRNA complexes

The free energies of binding were calculated from simulations through the Generalized Born and surface area continuum solvation (MM-GBSA) methodology. MM-GBSA has been used for the prediction of the binding energies of protein–small ligand and nucleic acid–small ligand systems,^49^–^51^ since it offers a good compromise between efficiency and accuracy, providing relevant information on the distribution of the binding energies of comparable systems and rationales for energy differences.^52^ Multiple short simulations with different initial velocities were performed to maximize the conformational sampling. This approach has been routinely implemented.^49^,^52^–^55^

**Fig. 7:**
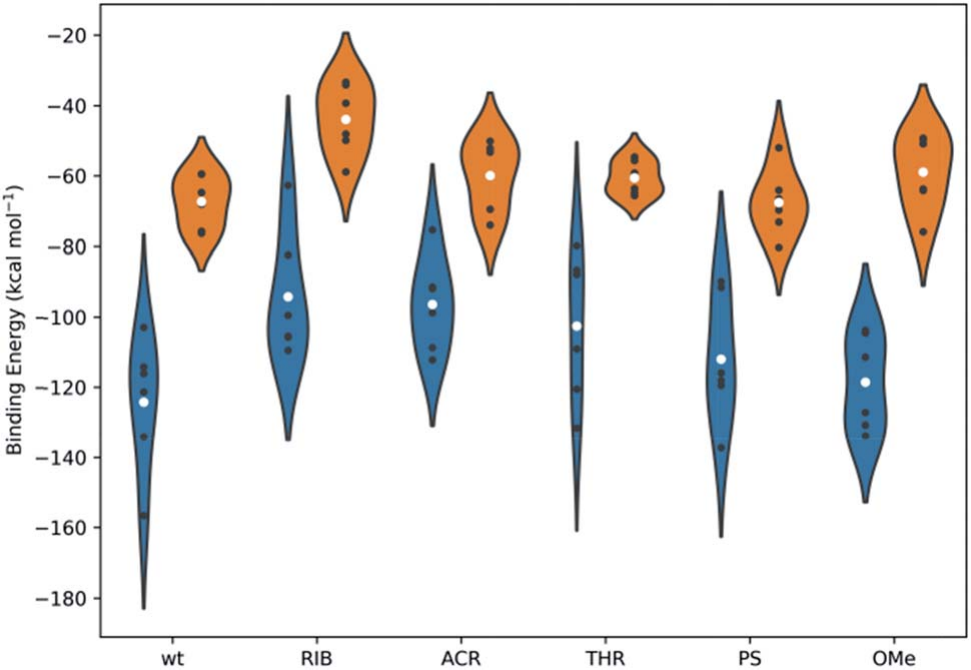
The binding energies, calculated using the MM-GBSA methodology, for all systems under study. The white dots represent the mean values of the free energy, calculated from 6 independent MD simulations (the values represented as black dots). The kernel distributions, represented by different colors, correspond to the estimated statistical distribution of the binding energies. Blue denotes the simulations started from the crystal coordinates of PDB ID 1SI2 and orange corresponds to those started from the crystal coordinates of PDB ID ; 4F3T. Tables S5 and S6 in the ESI† summarize the specific values obtained individually for each simulation and for the averaged data compiled from the 6 replicates.

Fig. 7 presents the mean values of the free energies estimated through the MM-GBSA method and from the 6 replicated simulations of set I and set II. The data were computed for all the PAZ/3′-overhang complexes, considering the last 10 ns of the production runs. The binding free energies were further decoupled into the individual components (see Tables S5 and S6†).

From Fig. 7, it is suggested that the interaction between PAZ and siRNA is an energetically favorable process (Δ*G*_bind_ < 0) for all chemical modifications. It should be noted that different crystal structures (set I and set II) show binding energies that follow comparable trends, although they present shifted absolute values.

In set I, the positioning of the dT and OMe analogues in the last 2-nt of the 3′overhang displays high affinity to the PAZ domain, with mean values of –124 and –119 kcal mol^–1^, respectively. Slightly lower affinity (Δ*G*_bind_ = –112 kcal mol^–1^) is shown when PS is used. In an opposite trend, the inclusion of nucleic acid derivatives either destitute of any nucleobase (RIB) or with a bulky nucleobase modification (ACR), combined with a deep modification of the ribose ring, reduces the binding affinity (RIB: Δ*G*_bind_ = –94 kcal mol^–1^ and ACR: Δ*G*_bind_ = –96 kcal mol^–1^). In the case of substitution of the 3′-overhang with THR, an intermediate strength of binding to the PAZ domain is observed, with Δ*G*_bind_ = –103 kcal mol^–1^. In set II, all the modifications proved to bind effectively to the PAZ domain following the same trend of set I (wt: Δ*G*_bind_ = –67 kcal mol^–1^, PS: Δ*G*_bind_ = –68 kcal mol^–1^, THR: Δ*G*_bind_ = –61 kcal mol^–1^, ACR: Δ*G*_bind_ = –60 kcal mol^–1^, OMe: Δ*G*_bind_ = –59 kcal mol^–1^ and RIB: Δ*G*_bind_ = –44 kcal mol^–1^), with the exception of OMe, for which the binding affinity of Ago2 to PAZ is lower. The binding energies for PAZ/3′-overhang siRNA are in good agreement with the experimental data (see Table 1). The MD results also suggest similar low binding affinities for ACR and RIB. The stronger binding associated to dT, PS, OMe and THR seems to dictate their higher potency of silencing (dT: IC_50_ = 6.3 pM, PS: IC_50_ = 5.1 pM, OMe: IC_50_ = 4.4 pM, THR: IC_50_ = 3.3 pM), while the weaker affinities of PAZ to RIB and ACR (RIB: IC_50_ = 34.1 pM, ACR: IC_50_ = 7.7 pM) are reflected by the respective lower values of the RNAi activity.

The energy differences between set I and II can be correlated with the level of structural complexity of the siRNA molecules, *i.e.*, the distinct RNA secondary structures and chain lengths. The Δ*G*_bind_ values obtained for set I have a wide dispersion, although this is smaller in set II. This results in the sampling of a larger region of the configuration space for MD trajectories of the same overall length; this is supported by the RMSD profiles (Fig. S1 and S2†). The higher number of siRNA nucleotides in set I promotes an increase in the degrees of freedom upon binding and, thus, a higher fluctuation. Furthermore, while the MM-GBSA binding energies may have significant absolute errors, the relative energies between similar compounds binding the same host are much more accurate.^56^–^58^ This also explains the large shift between the two crystal structures, despite following the same trend.

The decomposition of the binding free energy into different components (see eqn (S2)†) gives additional information about the nature of the interaction. Δ*G*_bind_ was calculated as the sum of the gas-phase energies, including the coulombic and van der Waals energies, and solvation free energies. The electrostatic contribution was calculated using GB theory, a non-polar part calculated using the solvent accessible surface area, and the translational entropy, including the rotational and vibrational terms. Focusing initially on data compiled for set I (Table S5†), it can be seen that, irrespective to the modified siRNAs, favorable Δ*G*_nonpol_ are obtained, suggesting that non-polar and hydrophobic interactions are relevant components for complex formation. An additional contribution of electrostatics is also identified, but to a lower extent (low negative values for Δ*G*_pol_). Δ*G*_pol_ for ACR modification is unfavorable, while Δ*G*_nonpol_ remains close to the value found for the unmodified counterpart, as a direct consequence of the partially hydrophobic character of the ACR moiety. The threoninol backbone is also shown to hamper the electrostatic contribution, considering the reduced contribution of the Δ*G*_pol_ calculated for both the THR and ACR analogues.

Set II (Table S6†) shows the same behavior for the energetic contributions, but for these complexes polar interactions become almost always unfavorable. In fact, the direct intermolecular electrostatic interactions, Δ*E*_ELE_, are favorable, as in set I, but they are largely surpassed by the desolvation penalties upon binding.

### Different binding modes in complexes of PAZ bound to modified 3′-overhang siRNA are found

To inspect in detail the contribution of the structural and chemical features of modified 3′-overhang in PAZ binding, we conducted a characterization of the most contributive residues in the complex interface by means of per-residue decomposition analysis. In addition, hydrogen bond analysis was also performed. The values obtained for per-residue decomposition using MM-GBSA method are summarized in Tables S7 (set I) and S8 (set II).† Taken together, the analysis of the per-residue free energy and H-bonds allows the complete identification of the residues involved in PAZ–siRNA interaction.

For set I (Table S7†), the residues that stand out for their contribution in the formation of the PAZ/siRNA wt complex are the basic K264, R275, K276, R278, K313, and K333 and the hydrophobic M273, all with Δ*G*_bind_ significantly lower than –2 kcal mol^–1^. This is observed in all systems, with some fluctuations in the respective intensity, as may be anticipated by the differences in Δ*G*_bind_. All these residues seem to be involved through hydrogen bonds/salt bridges (distance ≤ 3.5 Å) in the anchorage of siRNA, as represented in Fig. S3A† and establish interactions mainly with the intermediate residues of siRNA, UAG. Specifically, the hydrophobic M273 was found to interact with the sugar ring of the 5′-end of the siRNA complementary strand. The aromatic ring of F292 further contributes to the AS anchorage, to a lower extent, by interacting with the nucleobase of the last nt, dT3′. Less relevant in terms of the individual free energy contribution is an ensemble of other proteic residues, mainly located in the PAZ binding pocket that collectively strongly interacts with the last 2-nt of siRNA. By inspecting the per-residue free energy and H-bond behavior in set II, a high similarity between the two sets of simulations was found (Table S8 and Fig. S3B†). As observed in set I, the short AS of siRNA is attached to PAZ by the cooperative action of the H-bonds (not discarding also the possibility of salt bridge formation), Fig. S3B,† in which R277, Y311 and K335 show high relevance, as confirmed by the individual free energies (Table S8†). The range of free energies calculated to the last and second-last thymidine, varying from –4 to –15 kcal mol^–1^, displays good agreement with the Δ*G*_bind_ values, obtained by ITC measurements, for the PAZ domain from hAgo2 complexed to deoxynucleosides monophosphate.^41^

**Fig. 8:**
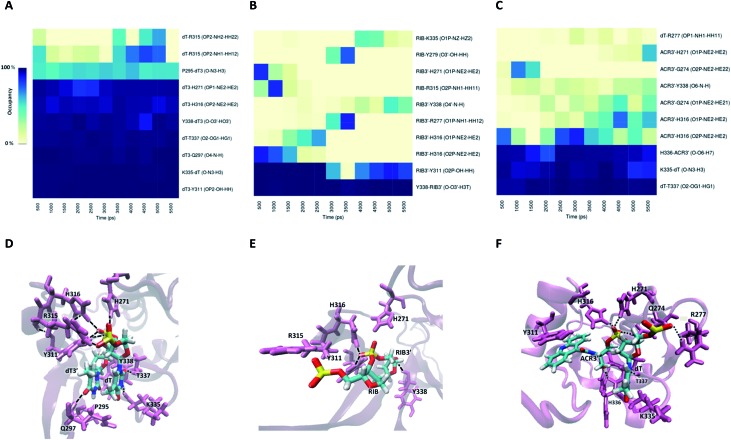
The interaction of the PAZ domain from hAgo2 with the modified siRNAs. Panels (A)–(C) represent the occupancies of the most prominent hydrogen bonds formed between the PAZ binding pocket and 2-nt modified nucleotides in the siRNA 3′-overhang, dT–dT3′, RIB–RIB3′, and dT–ACR3′, respectively. The data represented correspond to the last 11 ns from one MD simulation arbitrarily selected from the 6 MD replicas. The best representative snapshots of the complexes modified with dT–dT3′, RIB–RIB3′ and dT–ACR3′ are illustrated in panels (D), (E), and (F), respectively. The phosphorus atoms are colored in yellow, oxygen atoms in red, nitrogen atoms in blue, and hydrogen atoms in white. The protein side chains are represented in violet as cartoons and the siRNA bound amino acids are highlighted and shown as thin lines. The black dash lines represent the hydrogen-bonding interactions between the last two RNA nucleotides and the PAZ amino acid residues.

Information about the underlying H-bonds in the interface of the complex was obtained by the analysis of the instantaneous bond-formation using an in-house algorithm, leading to the representation of H-bond prevalence during the last nanoseconds of production run. Fig. 8A shows the most prevalent H-bonds in complex PAZ/siRNA-dT–dT3′ (set II), which display a pattern compatible with the individual binding affinities reported in Table S8.† Some interactions are found to be dynamic, with variable lifetimes in the range of picoseconds, confirming that adequate sampling was achieved. The recognition of the siRNA 3′-overhang by the PAZ domain of Ago2 is guided by the interaction of electronegative oxygens from the phosphodiester group and the amide groups of H271, H316, and R315, and by the carbonyl group of Y311 (Fig. 8D). Also, the terminal 3′-hydroxyl group of dT3′ binds to Y338. The last two 3′-overhang residues are oriented in parallel and well anchored to the hydrophobic amino acid P295 and polar Q297, which are located in β4, and to the charged amino acids K335, T337 and Y338 located in β7 of PAZ. This structural orientation of unmodified RNA is in good agreement with crystal data.^18^

The RIB and ACR modifications reduce the intermolecular contact, maintaining the interaction solely by two or three stable hydrogen bonds, and contact with β4 is avoided (Fig. 8B and C). If the nucleobase is removed from the nucleoside, as in RIB, contact between PAZ and siRNA is preferentially established by RIB3′ and the protein residues Y311 and Y338. With ACR, contact is preferentially made with the protein residues located in the loop’s β2–β3 and β7, such as H316, K335, H336 and T337. ACR perturbs the β5 and a short part of HIII, as indicated by RMSF analysis (Fig. 6F).

**Fig. 9:**
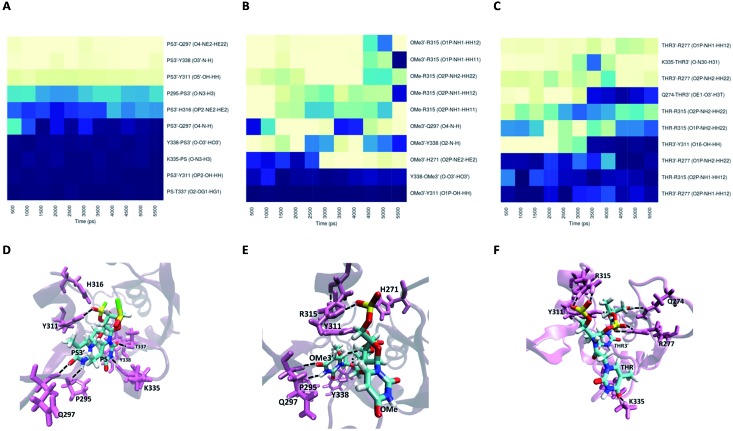
The interaction of the PAZ domain from hAgo2 with the modified siRNAs. Panels (A)–(C) represent the occupancies of the most prominent hydrogen bonds formed between the PAZ binding pocket and 2-nt modified nucleotides in the siRNA 3′ overhang, PS–PS3′, OMe–OMe3′ and THR–THR3′, respectively. The data represented correspond to the last 11 ns from one MD simulation arbitrarily selected from the 6 MD replicas. The best representative snapshots of the complexes modified with PS–PS3′, OMe–OMe3′ and THR–THR3′ are illustrated in panels (D), (E) and (F), respectively. The phosphorus atoms are colored in yellow, oxygen atoms in red, nitrogen atoms in blue, hydrogen atoms in white, and the sulphur atoms in green. The protein side chains are represented in violet as cartoons and the siRNA bound amino acids are highlighted and featured in thin lines. The black dash lines represent the hydrogen-bonding interactions between the last two RNA nucleotides and the PAZ amino acid residues.

For modification with PS, the contributions of the amino acids are quite similar to the ones found for unmodified siRNA (Fig. 9A), indicating a negligible impact of this modification on the interaction with PAZ. The PS groups reside in the same positions as the equivalent phosphates in siRNA with dT, but less H-bonds are formed, due to the presence of sulphur atoms. The simulation of siRNA modified with OMe presents less ability to form H-bonds (Fig. 9B). The loss of H-bonds and reduction of Δ*G*_bind_ consistently demonstrate the same trend. The complexes formed between PAZ/siRNA modified with THR possess a moderate number of H-bonds that occur in a transient mode (Fig. 9C). Resembling ACR and RIB, THR3′ does not interact with β4.

In the case of set I (see Fig. S4 and S5†), for complex PAZ/unmodified siRNA, the bonds linking the non-bridging oxygens of the phosphodiester backbone of the last nt and the polar AAs, H269 and Y309, and the linking between the terminal 3′-hydroxyl group and Y336 are among the more stable H-bonds. In addition, the sugar ring of dT3′ is tightly anchored to Q295 and Y336. With shorter lifetimes, other H-bonds are established between the phosphodiester backbone of dT3′ and the adjacent amino acids, K313 and Y314. The second-last nt, dT, is tethered to the binding pocket through interaction with Y277. These findings are consonant with the hydrogen bonds identified in the crystal structure.^47^

The substitution with RIB and ACR, as observed in set II, minimizes the contribution of almost every protein residues (see Fig. S4B and C†). Specifically for the modification with OMe, and in contrast to set II, there is conservation of the pattern obtained for unmodified siRNA, but with a slight reduction in the number of H-bonds and the free energy contribution of the terminal residues (Fig. S5B and E, Table S7†). PS and THR reduce the number of H-bonds with PAZ cleft (Fig. S5A and C†). As found for set II, the low number of H-bonds detected for the PAZ/siRNA-THR–THR3′ complex displays a dynamic pattern.

## Discussion

The use of siRNA in therapeutics holds tremendous potential, but is still hampered by limiting bottlenecks, such as physical instability, low potency *in vivo*, and unwanted side effects arising from wrong strand selection. The chemical modification of siRNA is currently a prerequisite for the optimization of siRNA performance, aiming essentially to increase the siRNA silencing potency and reduce the SS-mediated off-targeting effects, *i.e.* increase specificity.

In this work, we performed a systematic study covering a complete set of chemical modifications at the 3′-overhang of siRNA to analyze in detail how modifications in these regions of SS and AS affect siRNA potency. Moreover, the study of the impact of the asymmetry of the siRNA strands was pursued to unravel how chemical modifications can be useful to circumvent siRNA poor specificity. To explore these questions, the siRNA molecules included in this study were carefully designed to include chemical modifications involving unique physico-chemical properties, such as a lack of nucleobase, enantioselectivity, and steric hindrance, among others. Some of the modifications used, such as OMe and PS, are known to increase the siRNA potency and resistance to nucleases and are reportedly effective and fairly tolerated irrespective of their positioning in the siRNA chains.^47^,^59^,^60^ Abasic nucleotides have been incorporated in siRNA molecules both in external and internal positions and, contradictorily, shown to be detrimental or beneficial for siRNA potency.^9^,^61^,^62^ Interestingly, the presence of this analogue at some of the seed region positions prevents miRNA-like off-target effects.^9^,^62^ The study of bulky derivatives is also documented, but the compound synthesized in this work was never studied before in detail.^63^ The THR analogue has been explored in our lab recurrently due to its good silencing performance.^38^,^64^ The unusual analogues MIR and GNA were newly incorporated at the siRNA 3′ overhangs.

The results revealed that the introduction of an abasic residue, RIB, in the 3′-overhang of AS caused the most drastic reduction of the on-target activity and concomitantly induced the lowest repression of the SS-mediated off-targeting effects detected among all modifications. On the other hand, the modification with acyclic derivative, THR, resulted in the most effective increase in the silencing potency and efficiently reduces the SS-mediated off-targeting effects. As expected, OMe and PS modifications enhance the siRNA silencing effect compared to the siRNA wild type, while the modifications GNA and MIR maintain or produce a decline in the gene silencing activity. These results indicate the existence of a negative effect in siRNA potency if the nucleobase is either removed (RIB) or increased its steric hindrance (ACR) or even substituted by an enantiomer (MIR). On the other hand, modifications in the sugar (GNA, THR, and OME) and phosphodiester (PS) backbones tend to increase or at least maintain the siRNA potency. By the comparison of THR and GNA, in terms of the siRNA activity, a positive effect arising from a larger distance between the phosphodiester backbone and nucleobase is observed.

When SS is modified instead, all the siRNAs are equivalently potent in silencing, although the SS-mediated silencing is highly restricted only for modifications RIB and ACR. If the asymmetry of the siRNA molecules is further increased, holding one 3′-overhang chemically modified and the opposite 3′-overhang blunt-ended, results further underpin the importance of the 3′ regions. Therefore, having the AS of siRNA chemically modified and the complementary SS blunt-ended largely enhances the specificity of silencing. The use of blunt-ended overhangs has been demonstrated to have a negative effect in RISC loading and can predominantly increase the selection of overhang-containing strands.^65^–^67^ Again, the modification of AS with THR in the 3′-overhang is shown to be the most powerful modification in reducing SS-mediated silencing, especially when SS is blunt-ended. Interestingly, OMe is always ineffective in controlling the unwanted SS-mediated silencing.

The effect of the presence of non-active siRNA molecules indicated the negative contribution of a crowded and competitive environment to siRNA silencing. Specifically, non-active competitors holding 3′-overhang causes a higher drop in siRNA activity than blunt-ended ones, stressing the importance of 3′ protuberances on the success of siRNA activity. On the other hand, if the non-active competitor is in turn not able to be loaded into Agos, modified siRNAs still produce some decrease of silencing, pointing out possible RISC-independent mechanisms as the basis of siRNA silencing under these conditions. Interestingly, an siRNA holding bulky modification (ACR) is only modestly affected when exposed to any kind of competitive environment.

To fully understand the impact of the chemical modification of the 3′-overhang on siRNA activity, we studied separately the consequences of cancelling the action of some of the key players in the silencing process, the TRBP and Ago2 proteins. In the absence of a TRBP protein, which is essential to bridge DICER to the Ago2, the SS-mediated silencing for the siRNAs modified with RIB and ACR in AS is not affected, which is the opposite to what was observed for other modifications. This result might suggest that TRBP preferentially commits the sensing of siRNA asymmetry to the Ago2 protein. Consistent with this result is the finding that these same modifications are the only ones resulting in downregulation of the target mRNA in both the MEF and MEF Ago2^–/–^ cells. These remarkable findings provide new mechanistic insights into the modified siRNA activity, highlighting the dependency of the pathways followed during the RNAi process on the character of the modifications used.

On the basis of these experimental observations, we hypothesized that the interaction of the siRNAs 3′-overhangs with the PAZ domain plays a determinant role. To inspect this contribution we have carried out MD simulations. As such, the most relevant modified 3′-overhang siRNAs were modeled with the PAZ domain of both Ago1 and Ago2. From the MD results, we could devise a reliable description of complex PAZ/RNA either using different protein families (Ago1 and Ago2) or RNA substrates (single- or double-stranded), outlining the same overall trend for both families. Based on the structural analysis, a coherent picture emerges, in which the hampering of the H-bond formation in the active site of PAZ leads to reduction of the complex binding affinity. Thus, deeper structural modifications of the nucleotides, as made for RIB and ACR, present a detrimental effect in the PAZ/siRNA binding affinity, as well as a reduced number of H-bonds established with the PAZ binding pocket. RIB presents the weakest binding with the PAZ domain and the lowest silencing potency found experimentally. The positioning of one bulky nucleotide in the 3′-overhang impaired the efficient interaction with the hydrophobic cleft of the PAZ domain. The low binding affinities found computationally for RIB and ACR seem to establish a good correlation with the siRNA strand selection, in which an increment of the silencing specificity is promoted by a weak interaction between PAZ and the 3′-overhang of the SS. The subtle modifications, such as PS and OMe, moderately influence the accommodation of an siRNA 3′-overhang. Surprisingly, the OMe analogues present different abilities to interact with the Ago proteins, displaying a stronger binding to the PAZ domain of Ago1 than to the PAZ domain of Ago2. This difference might be correlated to the ability of the PAZ domains from distinct families to accommodate natural RNA modifications, as described in the literature.^68^,^69^ Despite the lower binding affinity of the synthetic siRNAs modified with OMe to PAZ from Ago2, this modification can strongly induce silencing exclusively *via* an Ago2-dependent mechanism, as outlined experimentally. Likewise, siRNAs modified with THR were found to bind moderately to the PAZ domain and experimentally they present the highest gene silencing efficacy. These observations indicate that a moderate binding between PAZ and the siRNAs is a requirement for the success of the RNAi activity, most likely to facilitate the consecutive dynamic steps previous to Ago slicing activity.

## Conclusions

The combined results presented here provide an overview into the dependency of the nature of the nucleotide analogues at the 3′-overhang on the siRNA potency and specificity. They also provide some hints on the propensity of some modifications to selectively downregulate mRNA following endonucleolytic cleavage and/or translational repression. The computational and experimental results hint at a direct connection between the PAZ/3′-overhang binding affinity and the siRNA potency/specificity. Thus, the higher RNAi activity is associated with a moderate-to-strong binding. On the other hand, lower binding energies may compromise the siRNA potency and favor siRNA downregulation by Ago2-independent mechanisms. A large enhancement in the avoidance of SS-mediated silencing can be achieved by modification of the SS 3′ overhang with a weaker binder to PAZ or simply using a SS that is blunt-ended. This work strongly demonstrates that the 3′-overhang is a key component of siRNA and highlights that the efficient modification of this terminus is a requirement to achieve more potent and specific siRNA-based therapeutics.

## Conflicts of interest

There are no conflicts of interest to declare.

## Supplementary Material

Supplementary information

## References

[cit1] Fire A., Xu S., Montgomery M. K., Kostas S. A., Driver S. E., Mello C. C. (1998). Nature.

[cit2] Elbashir S. M., Harborth J., Lendeckel W., Yalcin A., Weber K., Tuschl T. (2001). Nature.

[cit3] Wittrup A., Lieberman J. (2015). Nat. Rev. Genet..

[cit4] Alagia A., Eritja R. (2016). Wiley Interdiscip. Rev.: RNA.

[cit5] Stein C. A., Castanotto D. (2017). Mol. Ther..

[cit6] Ozcan G., Ozpolat B., Coleman R. L., Sood A. K., Lopez-Berestein G. (2015). Adv. Drug Delivery Rev..

[cit7] Ku S. H., Jo S. D., Lee Y. K., Kim K., Kim S. H. (2016). Adv. Drug Delivery Rev..

[cit8] Suter S. R., Sheu-Gruttadauria J., Schirle N. T., Valenzuela R., Ball-Jones A. A., Onizuka K., MacRae I. J., Beal P. A. (2016). J. Am. Chem. Soc..

[cit9] Lee H.-S., Seok H., Lee D. H., Ham J., Lee W., Youm E. M., Yoo J. S., Lee Y.-S., Jang E.-S., Chi S. W. (2015). Nat. Commun..

[cit10] Dua P., Yoo J. W., Kim S., Lee D.-k. (2011). Mol. Ther..

[cit11] Suter S. R., Ball-Jones A. A., Mumbleau M. M., Valenzuela R., Ibarra-Soza J. M., Owens H., Fisher A., Beal P. A. (2017). Org. Biomol. Chem..

[cit12] Valenzuela R. A., Onizuka K., Ball-Jones A. A., Hu T., Suter S. R., Beal P. A. (2016). ChemBioChem.

[cit13] Gu S., Kay M. A. (2010). Silence.

[cit14] Wilson R. C., Doudna J. A. (2013). Annu. Rev. Biophys..

[cit15] Shukla G. C., Singh J., Barik S. (2011). Mol. Cell. Pharmacol..

[cit16] Schirle N. T., MacRae I. J. (2011). Science.

[cit17] Wilson R. C., Tambe A., Kidwell M. A., Noland C. L., Schneider C. P., Doudna J. A. (2015). Mol. Cell.

[cit18] Elkayam E., Kuhn C.-D., Tocilj A., Haase A. D., Greene E. M., Hannon G. J., Joshua-Tor L. (2012). Cell.

[cit19] Parker J. S. (2010). Silence.

[cit20] Nakanishi K. (2016). Wiley Interdiscip. Rev.: RNA.

[cit21] Jackson A. L., Linsley P. S. (2010). Nat. Rev. Drug Discovery.

[cit22] Zander A., Holzmeister P., Klose D., Tinnefeld P., Grohmann D. (2014). RNA Biol..

[cit23] Jiang H., Sheong F. K., Zhu L., Gao X., Bernauer J., Huang X. (2015). PLoS Comput. Biol..

[cit24] Kandeel M., Kitade Y. (2013). PLoS One.

[cit25] Onizuka K., Harrison J. G., Ball-Jones A. A., Ibarra-Soza J. M., Zheng Y., Ly D., Lam W., Mac S., Tantillo D. J., Beal P. A. (2013). J. Am. Chem. Soc..

[cit26] Gaglione M., Potenza N., Di Fabio G., Romanucci V., Mosca N., Russo A., Novellino E., Cosconati S., Messere A. (2012). ACS Med. Chem. Lett..

[cit27] Lee H. S., Lee S. N., Joo C. H., Lee H., Lee H. S., Yoon S. Y., Kim Y. K., Choe H. (2007). J. Mol. Graph. Model..

[cit28] Anand R., Sanjeev B. (2016). J. Biomol. Struct. Dyn..

[cit29] Xia Z., Clark P., Huynh T., Loher P., Zhao Y., Chen H.-W., Rigoutsos I., Zhou R. (2012). Sci. Rep..

[cit30] Rashid U. J., Paterok D., Koglin A., Gohlke H., Piehler J., Chen J. C.-H. (2007). J. Biol. Chem..

[cit31] Wang Y., Li Y., Ma Z., Yang W., Ai C. (2010). PLoS Comput. Biol..

[cit32] Zhu L., Jiang H., Sheong F. K., Cui X., Gao X., Wang Y., Huang X. (2016). J. Phys. Chem. B.

[cit33] Kalia M., Willkomm S., Claussen J. C., Restle T., Bonvin A. M. (2015). Int. J. Mol. Sci..

[cit34] Xu L., Wang X., He H., Zhou J., Li X., Ma H., Li Z., Zeng Y., Shao R., Cen S. (2015). Biochemistry.

[cit35] Wang Y., Juranek S., Li H., Sheng G., Wardle G. S., Tuschl T., Patel D. J. (2009). Nature.

[cit36] Malefyt A. P., Wu M., Vocelle D. B., Kappes S. J., Lindeman S. D., Chan C., Walton S. P. (2014). FEBS J..

[cit37] Pascut D., Bedogni G., Tiribelli C. (2015). Biosci. Rep..

[cit38] Alagia A., Terrazas M., Eritja R. (2015). Molecules.

[cit39] Kamiya Y., Takai J., Ito H., Murayama K., Kashida H., Asanuma H. (2014). ChemBioChem.

[cit40] Strapps W. R., Pickering V., Muiru G. T., Rice J., Orsborn S., Polisky B. A., Sachs A., Bartz S. R. (2010). Nucleic Acids Res..

[cit41] Kandeel M., Al-Taher A., Nakashima R., Sakaguchi T., Kandeel A., Nagaya Y., Kitamura Y., Kitade Y. (2014). PLoS One.

[cit42] Bhandare V., Ramaswamy A. (2016). Adv. Bioinf..

[cit43] Deerberg A., Willkomm S., Restle T. (2013). Proc. Natl. Acad. Sci. U.S.A..

[cit44] Somoza Á., Terrazas M., Eritja R. (2010). Chem. Commun..

[cit45] Kawamata T., Tomari Y. (2010). Trends Biochem. Sci..

[cit46] Willkomm S., Restle T. (2015). Int. J. Mol. Sci..

[cit47] Ma J.-B., Ye K., Patel D. J. (2004). Nature.

[cit48] Nakanishi K., Ascano M., Gogakos T., Ishibe-Murakami S., Serganov A. A., Briskin D., Morozov P., Tuschl T., Patel D. J. (2013). Cell Rep..

[cit49] Wan S., Knapp B., Wright D. W., Deane C. M., Coveney P. V. (2015). J. Chem. Theory Comput..

[cit50] Henriksen N. M., Hayatshahi H. S., Davis D. R., Cheatham III T. E. (2014). J. Chem. Inf. Model..

[cit51] Krishnamurthy V. R., Sardar M. Y., Ying Y., Song X., Haller C., Dai E., Wang X., Hanjaya-Putra D., Sun L., Morikis V. (2015). Nat. Commun..

[cit52] Genheden S., Ryde U. (2015). Expert Opin. Drug Discovery.

[cit53] Wright D. W., Hall B. A., Kenway O. A., Jha S., Coveney P. V. (2014). J. Chem. Theory Comput..

[cit54] Adler M., Beroza P. (2013). J. Chem. Inf. Model..

[cit55] Sadiq S. K., Wright D. W., Kenway O. A., Coveney P. V. (2010). J. Chem. Inf. Model..

[cit56] Rastelli G., Rio A. D., Degliesposti G., Sgobba M. (2010). J. Comput. Chem..

[cit57] Hou T., Wang J., Li Y., Wang W. (2010). J. Chem. Inf. Model..

[cit58] Sun H., Li Y., Tian S., Xu L., Hou T. (2014). Phys. Chem. Chem. Phys..

[cit59] Harikrishna S., Pradeepkumar P. I. (2017). J. Chem. Inf. Model..

[cit60] Schirle N. T., Kinberger G. A., Murray H. F., Lima W. F., Prakash T. P., MacRae I. J. (2016). J. Am. Chem. Soc..

[cit61] Nagaya Y., Kitamura Y., Nakashima R., Shibata A., Ikeda M., Kitade Y. (2016). Nucleosides, Nucleotides Nucleic Acids.

[cit62] Liu J., Pendergraff H., Narayanannair K. J., Lackey J. G., Kuchimanchi S., Rajeev K. G., Manoharan M., Hu J., Corey D. R. (2013). Nucleic Acids Res..

[cit63] Aviñó A., Ocampo S. M., Perales J. C., Eritja R. (2012). Chem. Biodiversity.

[cit64] Alagia A., Terrazas M., Eritja R. (2014). Molecules.

[cit65] Song J.-J., Jidong Liu J.-J., Tolia N. H., Schneiderman J., Smith S. K., Martienssen R. A., Hannon G. J., Joshua-Tor L. (2003). Nat. Struct. Biol..

[cit66] Sano M., Sierant M., Miyagishi M., Nakanishi M., Takagi Y., Sutou S. (2008). Nucleic Acids Res..

[cit67] Ghosh P., Dullea R., Fischer J. E., Turi T. G., Sarver R. W., Zhang C., Basu K., Das S. K., Poland B. W. (2009). BMC Genomics.

[cit68] Simon B., Kirkpatrick J. P., Eckhardt S., Reuter M., Rocha E. A., Andrade-Navarro M. A., Sehr P., Pillai R. S., Carlomagno T. (2011). Structure.

[cit69] Ameres S. L., Horwich M. D., Hung J.-H., Xu J., Ghildiyal M., Weng Z., Zamore P. D. (2010). Science.

