# Supplementary material for: Exploring PAZ/3′-overhang interaction to improve siRNA specificity. A combined experimental and modeling study
Source: Chem Sci. 2018 Jan 15;9(8):2074–86. doi: 10.1039/c8sc00010g (PMC5896489; doi:10.1039/c8sc00010g)
Supplement: Supplementary file 1 [file SC-009-C8SC00010G-s001.pdf]

Electronic Supplementary Material (ESI) for Chemical Science.
This journal is © The Royal Society of Chemistry 2018

Supporting Information

Exploring PAZ/3'-overhang interaction to improve siRNA specificity. A combined experimental and modeling study

Adele Alagia, *[ab] ‡ Andreia F. Jorge,*[c] ‡ Anna Aviñó,[ab] Tânia F. G. G. Cova,[c] Ramon Crehuet,[a] Santiago Grijalvo,[ab] Alberto A. C. C. Pais[c] and Ramon Eritja*[ab]

[a.] *Institute for Advanced Chemistry of Catalonia (IQAC-CSIC), Jordi Girona 18-26, E-08034 Barcelona, Spain.*
[b.] *Networking Center on Bioengineering, Biomaterials and Nanomedicine (CIBER-BBN), Jordi Girona 18-26, E-08034 Barcelona, Spain.*
[c.] *CQC, Department of Chemistry, University of Coimbra, Rua Larga, 3004-535 Coimbra, Portugal.*
‡ Co-first authors with equal contribution to this work.
* Corresponding authors. Email: recgma@cid.csic.es; adele-alagia@iqac.csic.es; andreiaj@qui.uc.pt; Phone +34934006145

Table of contents:

1. **Materials and Methods** **Pages 2-6**
2. **MD results** **Pages 6-16**
3. **References** **Page 17**

1. Materials and Methods

1.1. Experimental methods

1.1.1 RNA synthesis and siRNA preparation

Modified RNA strands were synthesized on the 0.2-μmol scale using LV200 polystyrene supports. 3'- modified RNAs were synthesized on the 1-μmol scale using CPG functionalized with β-L-Thymidine,[1] L-threoninol-Acridine,[2] 2-deoxyRibitol[3] and GNA-Thymine[4] units as solid supports. All oligonucleotides were synthesized on an Applied Biosystems 3400 synthesizer using commercially available reagents and 2'-O-TBDMS-5'- O-DMT-protected phosphoramidites (A^{Bz}, G^{dmf}, C^{Ac} and U) in DMT-ON mode. The coupling time was 15 min and the coupling yields of natural and modified phosphoramidites were >97%. Unmodified (**wt**, **19**, **SCR** and **BLT**) and phosphorothioate (**PS**) oligonucleotides (**Table S1 and Table S2**) were purchased from Sigma-Aldrich. SiRNA sequences described by Terrazas *et al.*[5] were used to design siRNA duplexes against *Renilla* gene (**Table S2**) and non-active scrambled sequences (SCR and BLT) (**Table S3**). Solid supports were treated at 55 °C for 1 h with 1.5 mL of NH_3 solution (33%) and 0.5 mL of ethanol. Then, the oligonucleotides were purified using Glen-Pack™ RNA purification cartridges (Glen Research), quantified by absorption at 260 nm and analyzed by HLPC Column: Nucleosil 120–10 C18 column (250 × 4 mm). Solvent A: 5% ACN in 0.1 M aqueous TEAAc (pH = 7) and solvent B: 70% ACN in 0.1 M aqueous TEAA (pH = 7). Flow rate: 3 mL/min. Conditions: 20 min linear gradient from 15% to 80% B and 5 min 80% B. Finally, the oligonucleotides were confirmed by MALDI mass spectrometry (**Table S1**). SiRNA duplexes were prepared by annealing equimolar ratios of *sense and antisense siRNA strands* were in *siRNA suspension buffer* (100 mM KOAc, 30 mM HEPES-KOH, 2 mM $MgCl_2$, pH 7.4) to a final concentration of 20 μM, duplexes were then heated at 95 °C for 5 min and slowly cooled to 4 °C.

Table S1. RNA sequences used for the formation of siRNA duplexes.

**5'- AUCUGAAGAAGGAGAAAAA*XY* (SS)**<br>***ZW*UAGACUUCUUCCUCUUUUU -5' (AS)**				
	WZ-3' Antisense Strand (AS)	MW (Calculated) [Found]	*XY*-3' Sense Strand (SS)	MW (Calculated) [Found]
**wt**	dT; dT	(6439) [6434]	dT; dT	(6813) [6809]
**19**	----------	NA	------------	NA
**OMe**	OMeU; OMeU	(6475) [6475]	OMeU; OMeU	(6842) [6842]
**PS**	$_{PS}$dT; $_{PS}$dT	(6471) [6468]	$_{PS}$dT; $_{PS}$dT	(6845) [6844]
**RIB**	dRibitol; dRibitol	(6198) [6200]	dRibitol; dRibitol	(6565) [6568]
**ACR**	dT; Acridine	(6513) [6505]	dT; Acridine	(6879) [6874]
**MIR**	L-dT; L-dT	(6439) [6443]	L-dT; L-dT	(6813) [6809]
**GNA**	GNA-T; GNA-T	(6361) [6365]	GNA-T; GNA-T	(6729) [6732]
**THR**	L-thr-dT; L-thr-dT	(6497) [6492]	L-thr-dT; L-thr-dT	(6864) [6859]

dT= Thymidine; OMeU= 2'-O-methyl-uridine; $_{PS}$dT =Phosphorothioate ; dRibitol = 1,4-*anhydro*-2-deoxy-D-ribitol; Acridine= Acridinyl-L-threoninol; L-dT = β-L-2'-deoxythymidine; GNA-T = Thymine glycol nucleic acid; L-thr-dT = L-threoninol-thymine.

Table S2. Strand composition of siRNA molecules used in this study.

	**wt**	**wt4**	**wt5**	**OMe2**	**OMe3**	**OMe4**	**OMe5**	**PS2**	**PS3**	**PS4**	**PS5**
**AS**	wt	19	wt	OMe	wt	19	OMe	PS	wt	19	PS
**SS**	wt	wt	19	wt	OMe	OMe	19	wt	PS	PS	19

	**RIB2**	**RIB3**	**RIB4**	**RIB5**	**ACR2**	**ACR3**	**ACR4**	**ACR5**	**MIR2**	**MIR3**	**MIR4**
**AS**	RIB	wt	19	RIB	ACR	wt	19	wt	MIR	wt	19
**SS**	wt	RIB	RIB	19	wt	ACR	ACR	ACR	wt	MIR	MIR

	**MIR5**	**GNA2**	**GNA3**	**GNA4**	**GNA5**	**THR2**	**THR3**	**THR4**	**THR5**
**AS**	MIR	GNA	wt	19	GNA	THR	wt	19	THR
**SS**	19	wt	GNA	GNA	19	wt	THR	THR	19

1.1.2 Cells

HeLa (ATCC), MEF (ATCC), MEF Ago2$^{-/-}$ (a kind gift of Dr. O'Carroll)[6] and TEF TRBP$^{-/-}$ cell lines (a kind gift of Dr. Gatignol)[7] were maintained in exponential growth in high-glucose Dulbecco modified Eagle medium (DMEM) (Gibco, Life Technologies) supplemented with 10% heat inactivated fetal bovine serum (Gibco, Life Technologies) and 1× penicillin/streptomycin solution (Gibco, Life Technologies, Carlsbad, CA, USA). All cell lines were incubated at 37 °C in a humidified environment with 5% CO_2 and periodically checked for the presence of mycoplasma contamination.

1.1.3 Psicheck2 AS and SS Reporters

Psicheck2 AS and SS vectors have been previously described.[8]

1.1.4 Transfection and Luciferase Assay

24 H prior transfection, HeLa cells were seeded in 24-well plates at density of 1x10^5 cells well^{-1}; MEF and TEF TRBP$^{-/-}$ were plated in 24-well plates at density of 0.8x10^5 cells well^{-1}.

At time of transfection, the growth medium was changed with fresh DMEM +10% FBS without Pen/Strep (500 µl well^{-1}).

For AS/SS-mediated silencing experiments (in Hela, MEF and TEF TRBP$^{-/-}$ cells), either psicheck2 AS or psicheck2 SS vectors (1 ug well^{-1}) were co-transfected with 1 nM of siRNA using Lipofectamine 2000 (Life Technologies) (1.3 µg well^{-1}).

For IC_{50} assessment, psicheck2 AS (1 µg/well) was co-transfected with crescent concentration of siRNA molecules (2 pM, 8 pM, 16 pM, 60 pM, 0.16 nM, 0.3 nM and 1 nM) using Lipofectamine 2000 (1.3 µg/well) in accordance with manufacturer's instructions.

For AS/SS-mediated silencing assays under non-active siRNA competitive environment, psicheck2 AS or psicheck2 SS vectors (1 µg/well) were co-transfected with 30nM of siRNA competitors (SCR = canonical scrambled sequence, BLT = 3' blunt-ended scrambled sequence, FREE = siGENOME RISC-Free (Dharmacon) using Lipofectamine 2000 (1.5 µg well^{-1}). After 4 h, cells were gently washed twice in phosphate buffer saline

(PBS)(Gibco, Life Technologies) and 500 µl of fresh DMEM +10% FBS without Pen/Strep was added. Then, 1 nM of siRNA complexed with Lipofectamine 2000 (1 µg well^{-1}) was added to cells. The siRNA-mediated silencing on Renilla expression was measured on lysates collected 24 h post-transfection using Dual-luciferase reporter assay system (Promega) and Glomax luminometer (Promega). The ratios of *Renilla* luciferase (hRluc) to *Photinus* luciferase (hluc+) protein activities were normalized to mock transfection and the mock activity was set as 100%.

1.1.5 Ago2-dependent Silencing Assay

MEF and MEF Ago2$^{-/-}$ cells were seeded in 24-well plates at a density of 0.8 × 10^5 cells well^{-1} 24 h before transfection. Psicheck2 AS reporter (1 µg well^{-1}) was co-transfected with 1nM of siRNA molecules using Lipofectamine 2000 (1.3 µg well^{-1}). 24 h post-transfection cells were collected for RNA extraction.

1.1.6 Isolation of RNA and RT-qPCR

Total RNA was extracted from MEF and MEF Ago2$^{-/-}$ with TRIzol reagent (Invitrogen). Then, isolated RNA was quantified by NanoDrop (Thermo Scientific) and 2.5 µg of each RNA sample was treated with DNase I (RNase free) (New England Biolabs) following the manufacturer's instructions. Reverse transcription reaction was performed on 0.5 µg of total RNA using Oligo (dT) Primer and Revertaid H minus RT enzyme (Thermo Scientific) according to the manufacturer's instructions. cDNA was subsequently diluted 4 times UltraPure DNase/RNase-Free distilled water (Invitrogen) and 1 µL of resulting cDNA to the Maxima Sybr Green/ROX qPCR Master Mix (Thermo Scientific). Real-time qPCR was accomplished in a total volume of 20 µL, following the manufacturer's instructions. The reference gene GADPH was used as the internal control. *Renilla* silencing was calculated and represented as $2^{-\Delta\Delta Ct}$ method. All primer pairs were purchased from Sigma-Aldrich and Primer-Blast was used as the primer designing tool.

1.1.7 Statistical Analysis

Statistical analysis was performed using GraphPad Prism software (GraphPad, San Diego, CA, USA). IC_{50} determination was performed using non-linear regression analysis (log [inhibitor] vs. normalized response).

Table S3. Sequences of siRNA duplexes used in competition assay.

	AS	SS
**SCR**	5'-UCCUUUCUUUCUUUCGAUATT	5'-UAUCGAAAGAAAGAAAGGATT
**BLT**	5'-UCCUUUCUUUCUUUCGAUA	5'-UAUCGAAAGAAAGAAAGGA

Table S4. Primers list used in this study.

	Forward	Reverse
**GAPDH**	5'-TGCACCACCAACTGCTTAG	5'-GATGCAGGGATGATGTTC
**hRluc**	5'-GGGCGAGAAAATGGTGCTTG	5'-GCCCTTCTCCTTGAATGGCT

1.2. MD simulations

1.2.1 Starting structures

The starting structures used to describe the interaction between PAZ domain and 3'-overhang of siRNA were obtained from Protein Data Bank (PDB), selecting (i) the structure of PAZ from human Ago eIF2c1 (Set I) bound to the double stranded 9-mer siRNA, (PDB code: 1SI2)[9] and (ii) the structure of human Ago2 (Set II) bound to 4-mer single stranded miRNA (PDB code: 4F3T).[10] From the latter, the atoms corresponding to PAZ domain and the described four terminal nucleotides belonging to 3' end of siRNA were selected.

1.2.2 Molecular systems construction

In the preparation of PAZ, the lacking residues were added to the initial crystal structure and optimized through Modeller9v15.[11] The N and C termini were capped with neutral acetyl and N-methyl group, respectively. Hydrogen atoms were added using Leap module of AMBER14. The overall charge of PAZ domain was set to correspond to pH=7. In comparison to experimental counterpart, the last two residues positioned in the 3'-end terminal of the RNA x-ray crystal structure, were substituted by the natural nucleotide thymidine or by chemically modified nucleotides (see **Table S1**). The remaining nucleotide sequence match the one used experimentally. Similarly to protein construction, the Leap module was used to modify siRNA molecules. The new modified residues were parameterized by using the R.E.D.D. server[12] which automates the calculation of RESP charges at the HF/6-31G(d) level of theory, consistent with the AMBER14SB force field. Specifically for 2'-O-methyl and phosphorothioate modifications additional force fields have been used.[13,14] Both phosphorothioate residues were simulated considering R_p configuration. All crystal water molecules surrounding PAZ/siRNA complex were considered during simulations.

1.2.3 Molecular dynamic simulations

PAZ/siRNA complexes were parameterized using AMBER's ff14SB force field, which collects the recent updates of the backbone and the χ torsion parameters.[15-17] The original complex conformations were initially minimized for 2500 cycles using the steepest-descent algorithm in implicit Generalized Born (GB) solvent, and the final geometries were then solvated in an octahedral box. All systems were solvated using a TIP3P waters[18] maintaining a minimum distance between the complex and the box wall of approximately 12 Å. For net charge neutralization sodium ions were added. In order to ensure conditions close to the experimental ones, Na^+ and Cl^- ions were added to the complex to attain a concentration of approximately 150 mM, and their positions were randomized with Amber's Ptraj program.[19] The number of solvent molecules added to each system was made to be identical within related crystal structures, allowing the energetic comparisons between siRNA modifications.

All the solvated systems were initially minimized in two steps. During initial step, the atom positions of the siRNA and PAZ molecules were restrained applying 25 Kcal mol^{-1} $Å^{-2}$ of positional restraints, using 1000 steps for the steepest-descent algorithm, followed by a conjugate-gradient minimization involving another 1000 steps. In the second step, the entire system was minimized without restraints using the same algorithms as the previous step but with 2500 steps for each. Subsequently, the systems were heated from 0 to 298 K using Langevin thermostat over 1 ns with collision frequency of 2 ps^{-1} and 5 Kcal mol^{-1} $Å^{-2}$ of positional restraints on all of the solute atoms. Final equilibration at constant pressure and at 300 K was carried out for 2 ns using Langevin thermostat, and using 0.5 Kcal mol^{-1} $Å^{-2}$ of positional restraints on the solute atoms. Finally, unrestrained systems were simulated under NPT ensemble conditions using periodic boundary conditions and particle mesh Ewald (PME)[20] for long range charge interactions. For all heating, equilibration and production runs, the time step was set to 2 fs, and short-range interactions were set to a cutoff of 10 Å. Bonds involving hydrogen were held fixed using the SHAKE algorithm.[21] System coordinates were recorded every 2 picoseconds. Six short simulations were produced from the crystal structure with different initial velocities, via random number generator seeds using the option ig = -1 of AMBER. Production simulations were carried out

using CPU version of PMEMD program in AMBER14[22] on Navigator computing cluster of LCA-UC.[23] VMD was used for visualization.[24] RSMD, RMSF and H-bond analysis were carried out in AMBER's Ptraj program. Data from H-bond analysis were compiled and processed using an in-house script developed by the authors using Fortran 77 and R programming.

The hydrogen bonds were considered relevant when the distance between acceptor-donor is equal or less than 3.5 Å and the angle cutoff of equal or higher than 135°. The last 15 ns of the trajectory were used for RMSF and H-bonds sampling.

1.2.4 Energy analysis using MM-GBSA

Generalized Born and surface area continuum solvation (MM-GBSA) method[25] is an end-state calculation frequently employed to estimate the binding free energy in a noncovalently bound protein-ligand complex. To obtain a detailed description of the PAZ/siRNA interaction, MM-GBSA was used for implicit solvation using Debye-Hückel screening. Following the MM-GBSA approach, the binding free energies were calculated according to:

$$\Delta G_{bind} = \langle G_{PAZ-siRNA}(x)\rangle - \langle G_{PAZ}(x)\rangle - \langle G_{siRNA}(x)\rangle \qquad \text{(S1)}$$

where each term is estimated from snapshots, denoted as (x), taken from MD trajectories. For each snapshot, the free energy of a state is estimated by:

$$G(x) = H_{gas}(x) + H_{trans/rot}(x) + G_{solvation}(x) - TS(x) \qquad \text{(S2)}$$

where the first two terms, $H_{gas}(x)$ and $H_{trans/rot}(x)$ are calculated from internal (bond, angle, dihedrals), electrostatic and van der Waals energies. The third term of the equation, $G_{solvation}(x)$ comprises the contribution of polar and non-polar terms in which the polar contribution is typically obtained by using the generalized Born (GB) model whereas the non-polar tern is estimated from the solvent-accessible surface area (SASA) determined with the LCPO method.[26] Finally, the last term, $TS(x)$ corresponds to the conformational entropy to the binding. MM-GBSA analyses were made with MMPBSA.py program[27] in AMBER14 using the last 5000 frames from the equilibrated trajectory with a single trajectory approach. Nmode calculations are particularly computationally demanding for large systems, such as protein-RNA, and the analysis was conducted by analyzing using 8 to 15 snapshots. For MM-GBSA, a level-set-based dielectric model was used with an ionic strength equal 150 mM, and a solvent probe of 1.4 Å radii. Using the same frames as those used in the binding free energy calculations; the interaction components for PAZ and siRNA complex were also determined for evaluating the individual binding free energy per-residue at complex interface. For multiple short simulations, it was considered the average values from the 6 independent simulations, uncorrelated data points. SEM values were simply computed, dividing the sample standard deviation by the square root of the total number of simulations.

2. MD Results

a) dT-dT3’

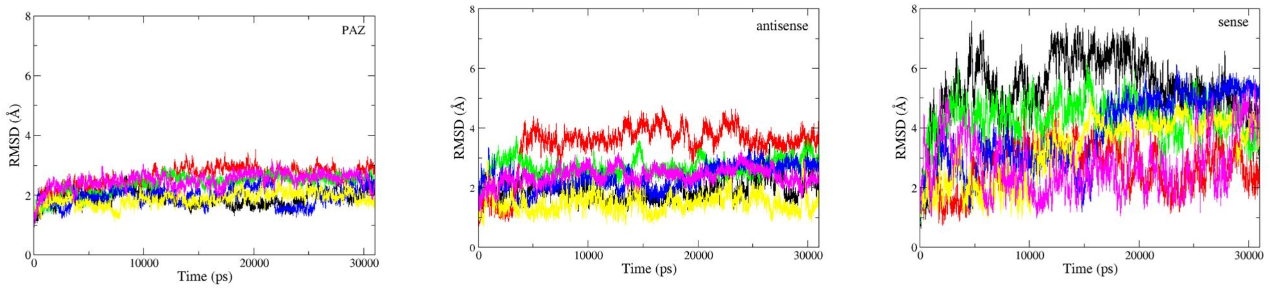

b) RIB-RIB3´

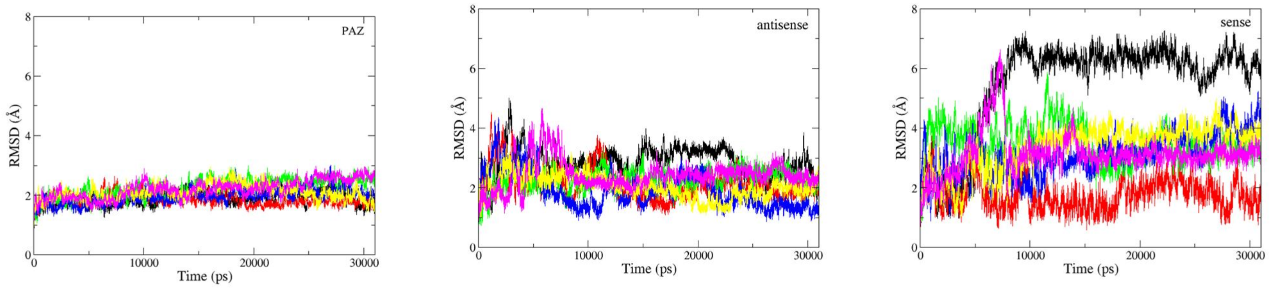

c) dT-ACR3’

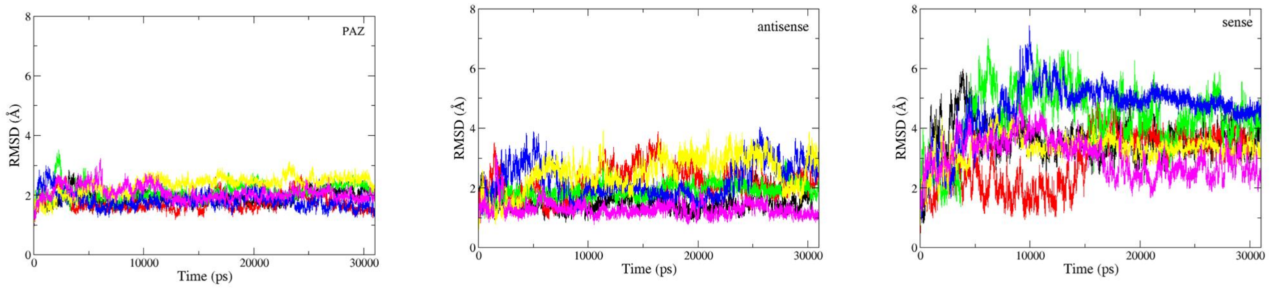

d) PS-PS3’

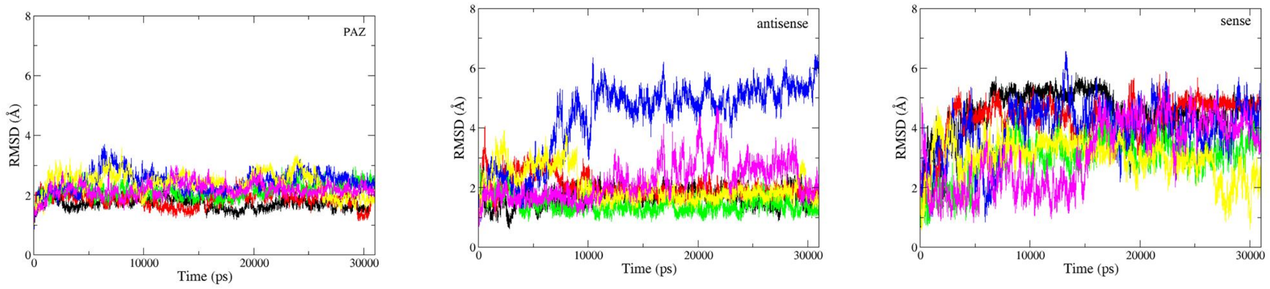

e) OMe-OMe3’

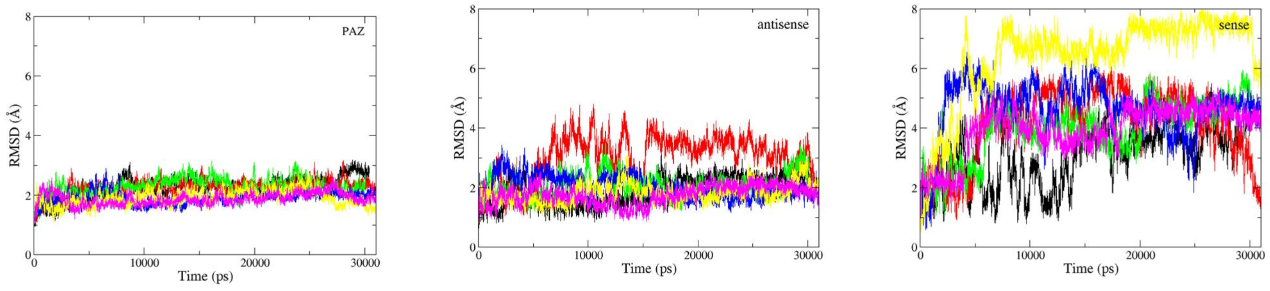

f) THR-THR3’

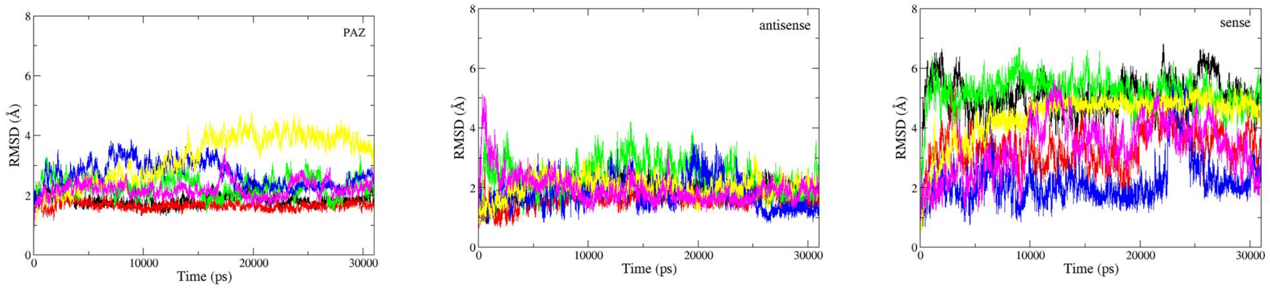

Figure S1. Comparison of the structural variations for complex PAZ/siRNA during the 31 ns of MD simulations for the siRNA with 2-nt 3’ overhang bearing (a) dT-dT3’, b) RIB-RIB3’, c) dT-ACR3’, d) PS-PS3’, e) OMe-OMe3’ and f) THR-THR3’. The complex initial coordinates were taken from PDB code 1SI2. The structural behaviour of PAZ, 9-mer siRNA antisense strand and 9-mer siRNA sense strand is shown separately. The heavy atoms, Cα atoms pertaining to PAZ backbone and from RNA heavy atoms (P, O3', O5', C3', C4', C5') were used for RMSD analysis and the first frame of production simulation was used as RMSD reference structure. Each RMSD curve corresponds to the 6 independent MD replicas.

a) dT-dT3’

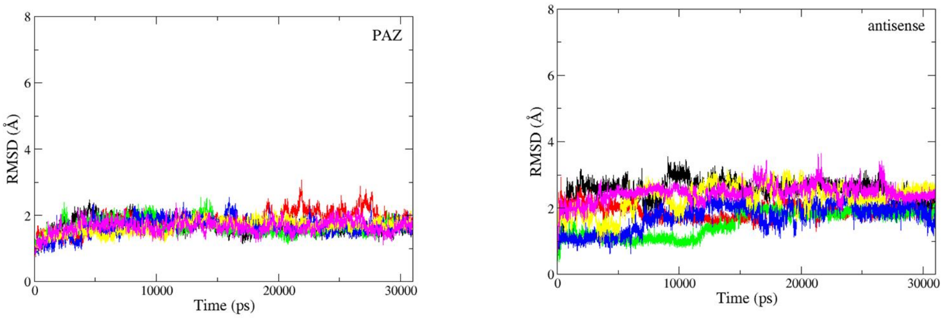

b) RIB-RIB3’

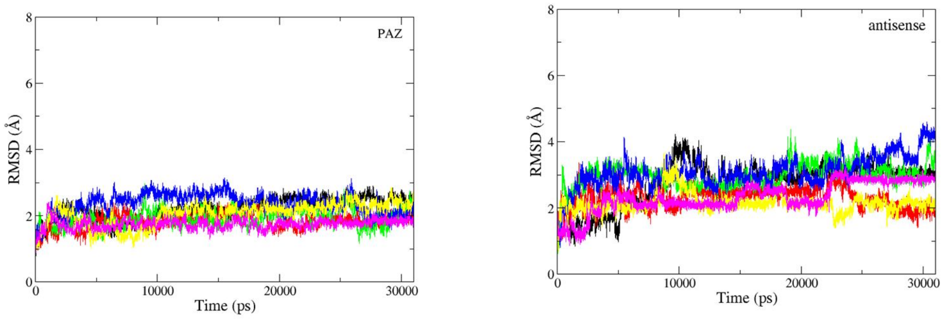

c) dT-ACR3’

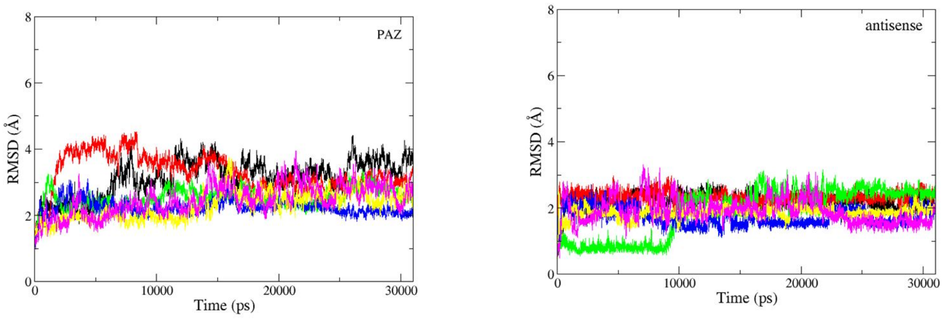

d) PS-PS3’

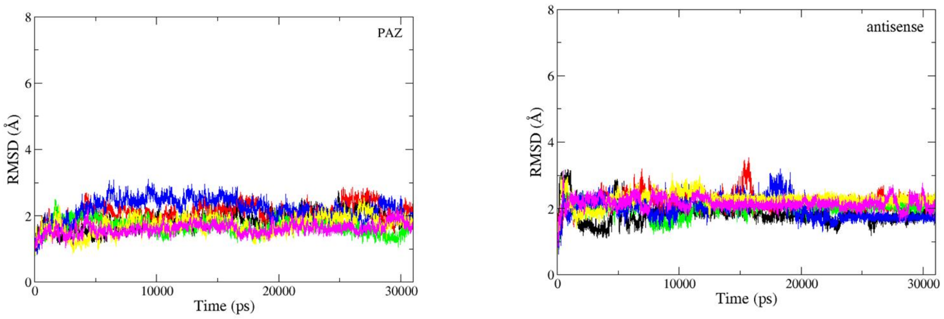

d) OMe-OMe3’

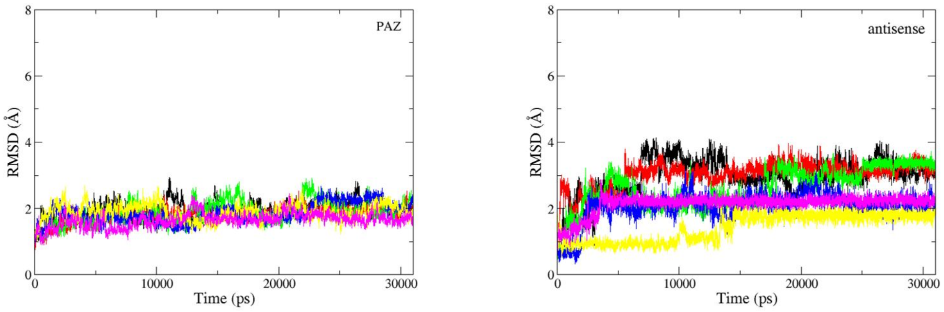

e) THR-THR3'

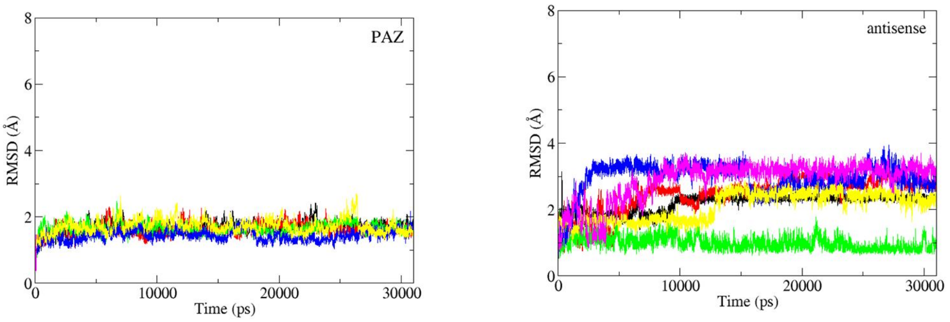

Figure S2. Comparison of the structural variations for complex PAZ/siRNA during MD simulations for the siRNA with 2-nt 3' overhang bearing (a) dT-dT3', b) RIB-RIB3', c) dT-ACR3', d) PS-PS3', e) OMe-OMe3' and f) THR-THR3'. The complex initial coordinates were taken from PDB code 4F3T. The structural behaviour of PAZ and 4-mer siRNA guide strand is shown separately. The heavy atoms, Cα atoms pertaining to PAZ backbone and from RNA heavy atoms (P, O3', O5', C3', C4', C5') were used for RMSD analysis and the first frame of production simulation was used as RMSD reference structure. Each RMSD curve corresponds to the 6 independent MD replicas.

Table S5. Binding free energy components of siRNA modified in 2-nucleotide 3' overhang complexed to PAZ of hAgo1 (PDB code: 1SI2) calculated from the last 5000 frames of 31 ns MD simulations. The values obtained for each simulation replica are compiled in this table and the mean values calculated. SEM values are also listed. All values are mentioned in kilocalories per mole and calculated using MM-GBSA methodology. Entropy contributions were calculated through normal-mode analysis over 8 snapshots.[a]

Systems	ΔE_{VDW}	ΔE_{ELE}	ΔE_{GB}	ΔG_{SA}	ΔG_{pol}[b]	ΔG_{nonpol}[c]	ΔG_{bind}[d]	TΔS	ΔG(ΔΔG)[e]
**dTdT3' – rep1**	-96	-2391	2344	-14	-47	-110	-157±11	-65±3	-92
**dTdT3' – rep2**	-75	-2368	2351	-11	-16	-87	-103±9	-57±6	-46
**dTdT3' – rep3**	-89	-2354	2323	-14	-32	-103	-134±13	-68±4	-66
**dTdT3' – rep4**	-89	-2343	2330	-14	-14	-102	-116±16	-60±8	-57
**dTdT3' – rep5**	-84	-2338	2321	-13	-17	-97	-114±11	-53±4	-62
**dTdT3' – rep6**	-93	-2348	2335	-14	-14	-108	-121±8	-60±5	-61
**dTdT3'-mean**	**-88±3**	**-2357±8**	**2334±5**	**-13±0.5**	**-23±5**	**-101±3**	**-124±8**	**-60±2**	**-64±8**
**RIBRIB3' – rep1**	-55	-1995	1997	-9	1.57	-64	-63±13	-46±4	-17
**RIBRIB3' – rep2**	-81	-2350	2337	-12	-13	-93	-105±11	-62±5	-44
**RIBRIB3' – rep3**	-56	-2137	2103	-11	-33	-66	-100±10	-64±6	-36
**RIBRIB3' – rep4**	-56	-2330	2313	-10	-17	-65	-82±10	-50±5	-32
**RIBRIB3' – rep5**	-72	-2393	2370	-11	-23	-83	-106±13	-49±4	-57
**RIBRIB3' – rep6**	-85	-2488	2478	-14	-10	-99	-110±11	-69±4	-41
**RIBRIB3'-mean**	**-67±6**	**-2282±74**	**2266±73**	**-11±0.7**	**-16±5**	**-74±6**	**-94±7**	**-54±4**	**-38±8**
**dTACR3' – rep1**	-95	-2153	2149	-13	-4	-109	-112±8	-65±3	-47
**dTACR3' – rep2**	-71	-2127	2117	-10	-10	-81	-92±9	-58±7	-33
**dTACR3' – rep3**	-98	-2047	2067	-13	20	-112	-91±8	-51±2	-40
**dTACR3' – rep4**	-80	-2191	2184	-11	-8	-91	-99±11	-54±3	-45
**dTACR3' – rep5**	-89	-2273	2267	-13	-6	-103	-109±10	-68±7	-41
**dTACR3' – rep6**	-81	-2109	2126	-12	17	-92	-75±9	-48±1	-27
**dTACR3'-mean**	**-86±4**	**-2150±31**	**2152±28**	**-12±0.5**	**2±5**	**-98±5**	**-96±5**	**-57±3**	**-39±6**
**PSPS3' – rep1**	-76	-2275	2270	-11	-5	-87	-92±11	-51±4	-40
**PSPS3' – rep2**	-89	-2493	2459	-13	-35	-102	-137±9	-57±3	-80
**PSPS3' – rep3**	-89	-2364	2347	-13	-17	-101	-119±13	-53±4	-66
**PSPS3' – rep4**	-95	-2480	2471	-14	-9	-108	-118±10	-68±3	-50
**PSPS3' – rep5**	-83	-2384	2363	-12	-21	-94	-116±8	-56±4	-60
**PSPS3' – rep6**	-74	-2146	2142	-11	-5	-85	-90±8	-56±4	-33
**PSPS3'-mean**	**-84±3**	**-2357±53**	**2342±50**	**-12±0.5**	**-15±5**	**-99±4**	**-112±7**	**-57±2**	**-55±8**
**OMeOMe3' – rep1**	-76.84	-2378.72	2335	-13	-44	-134	-134±8	-52±5	-82
**OMeOMe3' – rep2**	-75.66	-2258.82	2242	-11	-17	-104	-104±8	-50±1	-54
**OMeOMe3' – rep3**	-87.33	-2338.79	2327	-13	-11	-111	-111±8	-52±1	-60
**OMeOMe3' – rep4**	-92.70	-2404.23	2383	-14	-21	-127	-127±12	-56±5	-71
**OMeOMe3'– rep5**	-83.45	-2286.77	2278	-12	-9	-95	-105±11	-54±8	-51
**OMeOMe3' – rep6**	-78.03	-2555.87	2516	-13	-40	-91	-131±8	-53±5	-78
**OMeOMe3'-mean**	**-82.33±2.75**	**-2370.53±43.23**	**2347±39**	**-13±0.4**	**-24±6**	**-96±3**	**-119±6**	**-53±0.8**	**-66±6**
**THRTHR3'- rep1**	-71	-1967	1968	-10	1	-81	-80±8	-62±7	-18
**THRTHR3'- rep2**	-98	-2126	2129	-14	3	-112	-109±9	-58±4	-51
**THRTHR3'- rep3**	-78	-2116	2119	-11	3	-90	-87±10	-59±4	-27
**THRTHR3'- rep4**	-71	-2042	2036	-11	-6	-82	-88±23	-51±2	-37
**THRTHR3'- rep5**	-90	-2502	2475	-14	-27	-104	-132±11	-63±2	-68
**THRTHR3'- rep6**	-96	-2368	2357	-14	-11	-110	-121±12	-51±7	-69
**THRTHR3'- mean**	**-84±5**	**-2187±84**	**2181±80**	**-13±0.8**	**-6±5**	**-94±6**	**-103±9**	**-57±2**	**-45±9**

he symbols depicted in the table columns corresponds to: ΔE_{VDW}, van der Waals energy; ΔE_{ELE}, electrostatic energy; ΔE_{GB}, electrostatic contribution to the solvation free energy calculated by GB; ΔG_{SA}, non-polar contribution to the solvation free energy calculated with LCPO method; [b] $\Delta Gpol = \Delta E_{ELE} + \Delta E_{GB}$; [c] $\Delta G_{nonpol} = \Delta E_{VDW} + \Delta G_{SA}$; [d] $\Delta G_{bind} = \Delta E_{ELE} + \Delta E_{GB} + \Delta E_{VDW} + \Delta G_{SA}$; TΔS, total entropy contribution; [e] ΔG(ΔΔG) = ΔG – TΔS. ΔG(ΔΔG) is the total free energy.

Table S6. Binding free energy components of siRNA modified in 2-nucleotide 3' overhang complexed to PAZ of hAgo2 (PDB code: 4F3T) calculated from the last 5000 frames of 30 ns MD simulations. All values are mentioned in kilocalories per mole and calculated using MM-GBSA methodology and entropy contributions was calculated through normal-mode analysis over 15 snapshots.[a]

Systems	ΔE_{VDW}	ΔE_{ELE}	ΔE_{GB}	ΔG_{SA}	ΔG_{pol}	ΔG_{nonpol}	ΔG	TΔS	ΔG(ΔΔG)
dTdT3' – rep1	-60	-584	576	-8	-8	-68	-76±6	-33±5	-43
dTdT3' – rep2	-51	-612	594	-8	-18	-58	-76±9	-30±4	-46
dTdT3' – rep3	-62	-455	458	-8	3	-71	-68±7	-36±3	-32
dTdT3' – rep4	-61	-501	510	-8	9	-69	-60±5	-37±3	-23
dTdT3' – rep5	-55	-485	488	-7	3	-62	-60±7	-25±4	-34
dTdT3' – rep6	-50	-566	559	-7	-7	-58	-65±6	-39±3	-25
**dTdT3'-mean**	**-57±2**	**-534±25**	**531±22**	**-8±0.2**	**-3±4**	**-64±2**	**-67±3**	**-33±2**	**-34±4**
RIBRIB3' – rep1	-45	-474	477	-6	3	-51	-48±5	-37±4	7
RIBRIB3'– rep2	-43	-575	565	-6	-10	-49	-59±13	-36±2	13
RIBRIB3' – rep3	-25	-477	472	-4	-5	-29	-34±5	-20±4	7
RIBRIB3' – rep4	-32	-508	506	-5	-3	-37	-39±9	-32±5	-8
RIBRIB3' – rep5	-34	-552	542	-6	-10	-40	-50±7	-30±2	-18
RIBRIB3' – rep6	-30	-483	485	-5	2	-35	-33±7	-26±4	-7
**RIBRIB3'-mean**	**-35±3**	**-512±17**	**508±16**	**-5±0.4**	**-4±2**	**-40±3**	**-44±4**	**-29.94±2.57**	**-13±5**
dTACR3' – rep1	-59	-464	470	-8	7	-67	-60±5	-43±4	-17
dTACR3' – rep2	-47	-452	456	-6	7	-54	-50±6	-32±4	-18
dTACR3' – rep3	-72	-484	492	-9	7	-81	-74±7	-38±8	-36
dTACR3' – rep4	-59	-514	511	-8	-3	-67	-70±7	-33±5	-36
dTACR3' – rep5	-61	-458	474	-8	17	-69	-52±8	-35±4	-17
dTACR3' – rep6	-64	-429	448	-8	19	-72	-53±8	-37±3	-16
**dTACR3'-mean**	**-61±3**	**-467±12**	**475±10**	**-8±0.4**	**8±3**	**-68±4**	**-60±4**	**-36±2**	**-23±4**
PSPS3' – rep1	-68	-536	539	-9	4	-76.74	-73±7	-40±2	-34
PSPS3' – rep2	-63	-535	527	-8	-8	-71.94	-80±7	-40±4	-41
PSPS3' – rep3	-67	-538	543	-8	5	-74.93	-70±6	-35±2	-35
PSPS3' – rep4	-53	-504	500	-7	-4	-59.87	-64±8	-35±2	-29
PSPS3' – rep5	-49	-588	591	-7	4	-55.65	-52±6	-34±4	-18
PSPS3' – rep6	-55	-507	504	-8	-4	-62.78	-67±8	-38±1	-29
**PSPS3'-mean**	**-59±3**	**-535±12**	**534±14**	**-8±0.3**	**-0.65±2**	**-67±4**	**-68±4**	**-37±1**	**-31±4**
OMeOMe3' – rep1	-54	-470	480	-7	10	-61	-51±9	-28±6	-23
OMeOMe3' – rep2	-55	-553	551	-8	-2	-62	-64±7	-36±3	-28
OMeOMe3' – rep3	-63	-512	508	-8	-4	-71	-76±10	-34±4	-42
OMeOMe3' – rep4	-49	-466	473	-7	6	-56	-49±8	-26±6	-24
OMeOMe3' – rep5	-59	-417	420	-7	3	-67	-64±5	-34±2	-31
OMeOMe3' – rep6	-53	-439	494	-7	10	-60	-50±8	-33±3	-16
**OMeOMe3'-mean**	**-55±2**	**-484±19**	**488±18**	**-7±0.2**	**4±2**	**-63±2**	**-59±4**	**-33±1**	**-26±5**
THRTHR3'- rep1	-62	-476	481	-8	5	-70	-65±5	-35±2	-30
THRTHR3'- rep2	-55	-451	458	-7	6	-62	-56±6	-30±3	-26
THRTHR3'- rep3	-59	-428	435	-8	7	-67	-59±6	-33±3	-26
THRTHR3'- rep4	-53	-505	510	-7	5	-60	-55±9	-38±5	-17
THRTHR3'- rep5	-61	-514	520	-8	5	-69	-64±7	-37±4	-26
THRTHR3'- rep6	-59	-450	452	-8	2	-67	-66±8	-35±5	-31
**THRTHR3'- mean**	**-58±1**	**-471±14**	**476±14**	**-8±0.2**	**5±0.8**	**-66±2**	**-61±2**	**-35±1**	**-26±2**

[a]The symbols depicted in the table columns corresponds to: ΔE_{VDW}, van der Waals energy; ΔE_{ELE}, electrostatic energy; ΔE_{GB}, electrostatic contribution to the solvation free energy calculated by GB; ΔG_{SA}, non-polar contribution to the solvation free energy calculated with LCPO method; [b] $\Delta Gpol = \Delta E_{ELE} + \Delta E_{GB}$; [c] $\Delta G_{nonpol} = \Delta E_{VDW} + \Delta G_{SA}$; [d] $\Delta G_{bind} = \Delta E_{ELE} + \Delta E_{GB} + \Delta E_{VDW} + \Delta G_{SA}$; TΔS, total entropy contribution; [e] ΔG(ΔΔG) = ΔG – TΔS. ΔG(ΔΔG) is the total free energy.

Table S7. Per-amino acids and per-nucleotide decomposition free energy of hAgo1 and siRNA guide strand calculated from the last 5000 snapshots of MD simulations. All values are mentioned in kilocalories per mole and calculated by MM-GBSA approach. In these listed the values for residues with free energy higher than 2 kcal/mol in absolute value. Nucleotides correspond to siRNA guide strand.

Residues	dT-dT3'	RIB-RIB3'	dT-ACR3'	PS-PS3'	OMe-OMe3'	THR-THR3'
**K264**	-7±0.4	-7±0.9	-3±0.8	-6±0.7	-7±1	-5±0.6
**H269**	-3±0.6	-2±0.7	-1±0.8	-2±0.4	-4±0.1	-0.6±0.3
**M273**	-4±0.7	-3±0.3	-3±0.7	-4±0.5	-4±0.5	-4±0.5
**K274**	-3±0.4	-2±0.7	-3±0.5	-3±0.7	-3±0.7	-2±0.6
**R275**	-11±2	-11±1	-8±2	-14±1	-9±1	-10±3
**K276**	-5±1	-3±1	-2±0.6	-5±2	-0.9±0.5	-5±3
**R278**	-7±0.9	-6±2	-9±2	-4±2	-11±0.8	-6±2
**F292**	-3±0.2	-1±0.2	-2±0.4	-3±0.2	-2±0.4	-2±0.4
**Q295**	-2±1	-1±0.6	-0.3±0.2	-0.9±0.2	-0.2±0.2	-1±0.7
**Y309**	-4±0.1	-2±0.3	-1±0.4	-3±0.1	-3±0.3	-3±0.5
**K313**	-6±0.8	-5±0.8	-4±1	-5±0.5	-8±0.8	-6±2
**Q330**	-2±0.5	-3±1	-2±0.9	-1±0.3	-2±1	-2±0.9
**K333**	-6±1	-5±0.9	-6±0.9	-6±1	-7±2	-5±1
**T335**	-3±0.4	-2±0.2	-2±0.5	-3±0.3	-2±0.2	-1±0.6
**Y336**	-3±0.3	-2±0.2	-0.9±0.3	-3±0.3	-2±0.4	-0.7±0.4
**C**	-2±0.5	-3±0.7	-4±1	-2±0.8	-1±0.6	-2±0.7
**A**	-4±0.7	-3±1	-1±0.5	-4±1	-3±0.3	-3±1.1
**G**	-2±0.5	-2±0.9	-2±0.7	-3±1	-3±0.7	-2±0.7
**A**	-3±0.8	-3±0.3	-3±1	-6±1	-4±0.2	-3±1
**U**	-6±1	-3±0.7	-1±0.4	-3±0.5	-3±1	-3±0.6
**second-last nt**	-5±1	-3±1	-4±0.8	-3±0.4	-4±0.7	-8±2
**last nt**	-15±0.8	-9±1	-14±0.6	-13±0.4	-14±0.5	-9±0.6

Table S8. Per-amino acids and per-nucleotide decomposition free energy of hAgo2 and siRNA guide strand calculated from the last 5000 snapshots of MD simulations. All values are mentioned in kilocalories per mole and calculated by MM-GBSA approach. In these listed the values for residues with free energy higher than 2 kcal/mol in absolute value.

Residues	dT-dT3'	RIB-RIB3'	dT-ACR3'	PS-PS3'	OMe-OMe3'	THR-THR3'
H271	-3±0.6	-0.05±0.02	-0.8±0.3	-1±0.3	-1±0.6	-0.7±0.2
Q274	-1±0.5	-2±0.9	-2±0.4	-2±0.5	-3±1	-2±1
R277	-6±2	-7±1	-4±1	-7±2	-5±1.5	-5±1
F294	-3±0.1	-0.5±0.1	-3±0.8	-2±0.2	-2±0.2	-2±0.3
Y311	-3±0.2	-0.7±0.1	-2±0.3	-3±0.4	-4±0.3	-2±0.4
R315	-3±0.8	-4±0.8	-7±2	-3±2	-7±0.5	-6±1
H316	-3±0.4	-0.7±0.4	-2±0.5	-3±0.5	-3±0.5	-2±0.5
K335	-5±1	-6±2	-2±0.8	-3±0.9	-0.03±0.01	-3±0.9
T337	-3±0.1	-0.6±0.2	-1±0.6	-2±0.4	-2±0.1	-2±0.3
Y338	-3±0.1	-0.03±0.02	-0.3±0.1	-3±0.5	-2±0.3	-0.7±0.1
A	-3±0.7	-3±0.7	-4±0.8	-3±1	-2±0.7	-3±0.8
U	-2±0.5	-3±0.9	-4±0.8	-3±1	-2±1	-1±0.6
second-last nt	-7±0.3	-4±0.5	-6±2	-6±0.4	-6±0.5	-5±0.6
last nt	-12±0.6	-8±1	-12±2	-12±1	-12±1	-14±0.4

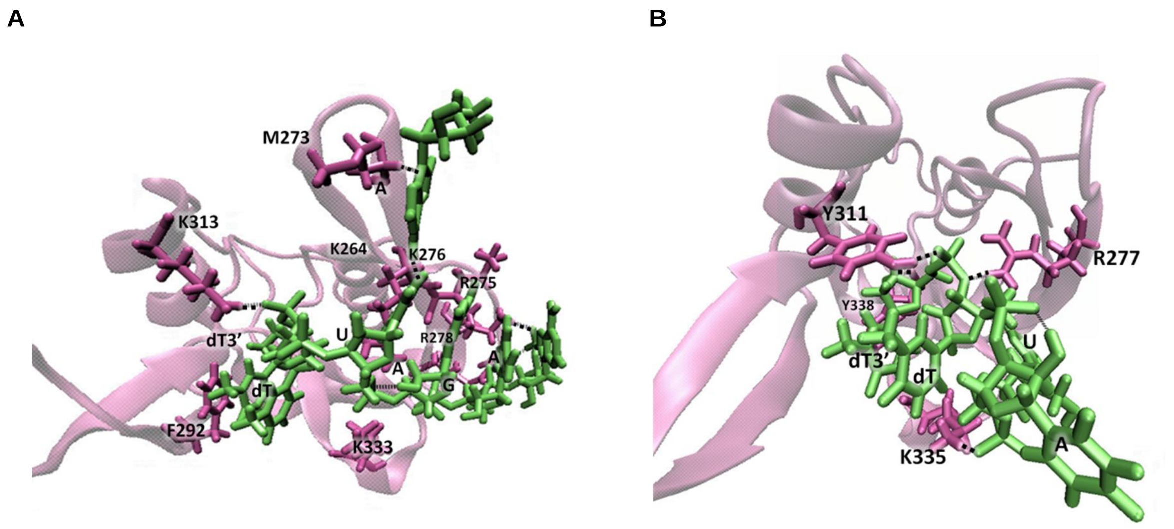

Figure S3. Best representative snapshots displaying hydrogen bonds and electrostatic interactions formed between A, PAZ domain from Ago1 and unmodified siRNA-like duplex and B, PAZ domain from Ago2 and unmodified 4-mer siRNA. Potential hydrogen bonds established between siRNA phosphodiester backbone and the basic PAZ residues are represented in dash lines and coloured in black. Protein side chains are represented in violet as cartoon and the siRNA bound amino acids are highlighted and featured in thin lines. RNA molecule is represented in green for simplicity.

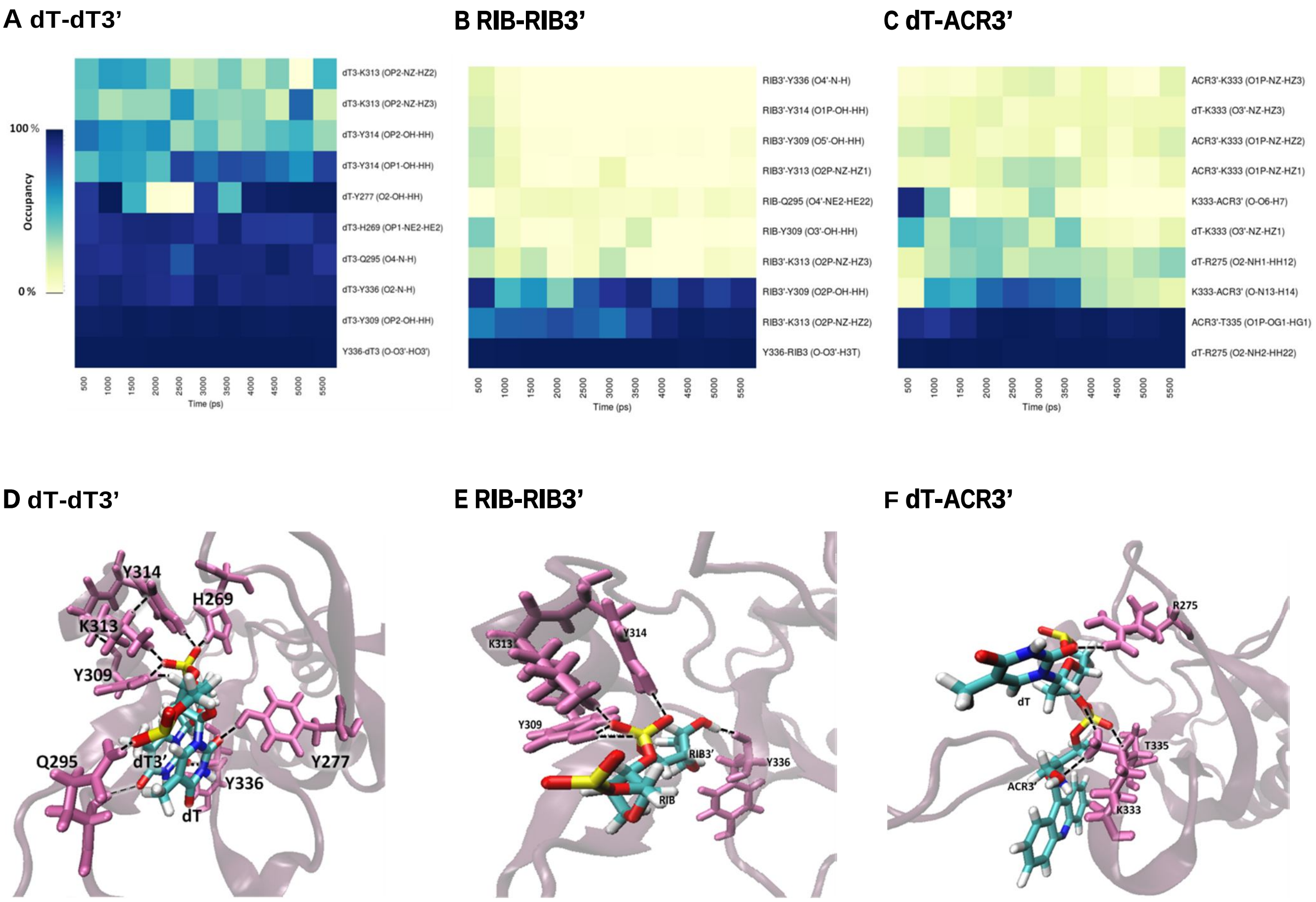

Figure S4. Interaction of PAZ domain from hAgo1 with modified siRNA-like duplex. In panels A and C are represented the occupancies of the most prominent hydrogen bonds formed between PAZ binding pocket and the 2-nt modified nucleotides in siRNA 3' overhang, dT-dT3', RIB-RIB3´ and dT-ACR3', respectively. Data represented correspond to the last 11 ns from one MD simulation arbitrarily selected from the 6 MD replicas. Best representative snapshots of complexes modified with dT-dT3', RIB-RIB3´ and dT-ACR3' are illustrated in panel D, E and F, respectively. Phosphorus are coloured in yellow, oxygens in red, nitrogens in blue and hydrogens in white. Protein side chains are represented in violet as cartoon and the siRNA bound amino acids are highlighted and featured in thin lines. The black dash lines represent the hydrogen-bonding interactions between the last two RNA nucleotides and the PAZ amino acid residues.

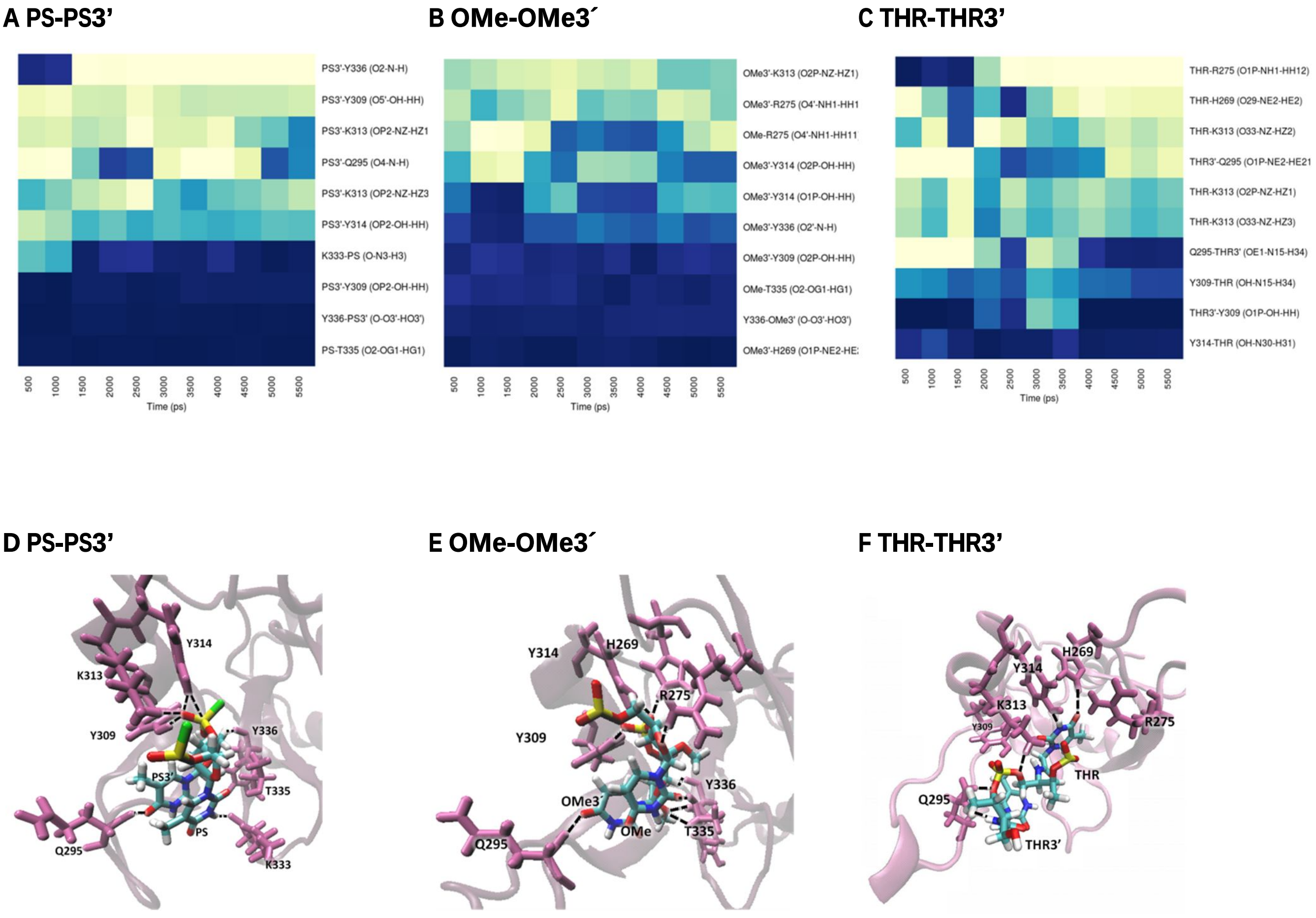

Figure S5. Interaction of PAZ domain from hAgo1 with modified siRNA-like duplex. In panels A and C are represented the occupancies of the most prominent hydrogen bonds formed between PAZ binding pocket and the 2-nt modified nucleotides in siRNA 3' overhang, PS-PS3', OMe-OMe3´ and THR-THR3', respectively. Data represented correspond to the last 11 ns from one MD simulation arbitrarily selected from the 6 MD replicas. Best representative snapshots of complexes modified with PS-PS3', OMe-OMe3´ and THR-THR3' are illustrated in panel D, E and F, respectively. Phosphorus are coloured in yellow, oxygens in red, nitrogens in blue, hydrogens in white and sulphurs in green. Protein side chains are represented in violet as cartoon and the siRNA bound amino acids are highlighted and featured in thin lines. The black dash lines represent the hydrogen-bonding interactions between the last two RNA nucleotides and the PAZ amino acid residues.

References

1. U. Asseline, J. F. Hau, S. Czernecki, T. Le Diguarher, M. C. Perlat, J. M. Valery and N. T. Thuong, *Nucleic Acids Res*, 1991, **19**, 4067-4074
2. A. Avino, S. Mazzini, R. Ferreira and R. Eritja, *Bioorg Med Chem*, 2010, **18**, 7348-7356.
3. V. Serebryany and L. Beigelman, *Nucleosides Nucleotides Nucleic Acids*, 2003, **22**, 1305-1307.
4. E. Meggers and L. Zhang, *Acc Chem Res*, 2010, **43**, 1092-1102.
5. M. Terrazas and R. Eritja, *Mol Divers*, 2011, **15**, 677-686.
6. D. O'Carroll, I. Mecklenbrauker, P. P. Das, A. Santana, U. Koenig, A. J. Enright, E. A. Miska and A. Tarakhovsky, *Genes Dev*, 2007, **21**, 1999-2004.
7. S. M. Daniels, C. E. Melendez-Pena, R. J. Scarborough, A. Daher, H. S. Christensen, M. El Far, D. F. Purcell, S. Laine and A. Gatignol, *BMC Mol Biol*, 2009, **10**, 38.
8. A. Alagia, M. Terrazas and R. Eritja, *Molecules*, 2015, **20**, 7602-7619.
9. J.-B. Ma, K. Ye and D. J. Patel, *Nature*, 2004, **429**, 318-322.
10. E. Elkayam, C.-D. Kuhn, A. Tocilj, A. D. Haase, E. M. Greene, G. J. Hannon and L. Joshua-Tor, *Cell*, 2012, **150**, 100-110.
11. B. Webb and A. Sali, in *Current Protocols in Bioinformatics*, John Wiley & Sons, Inc., 2002.
12. E. Vanquelef, S. Simon, G. Marquant, E. Garcia, G. Klimerak, J. C. Delepine, P. Cieplak and F.-Y. Dupradeau, *Nucleic Acids Research*, 2011, **39**, W511-W517.
13. K. E. Lind, L. D. Sherlin, V. Mohan, R. H. Griffey and D. M. Ferguson, in *Molecular Modeling of Nucleic Acids*, American Chemical Society, 1997, vol. 682, pp. 41-54.
14. R. Aduri, B. T. Psciuk, P. Saro, H. Taniga, H. B. Schlegel and J. SantaLucia, *Journal of Chemical Theory and Computation*, 2007, **3**, 1464-1475.
15. M. Zgarbová, M. Otyepka, J. Šponer, A. Mládek, P. Banáš, T. E. Cheatham and P. Jurečka, *Journal of Chemical Theory and Computation*, 2011, **7**, 2886-2902.
16. P. Banáš, D. Hollas, M. Zgarbová, P. Jurečka, M. Orozco, T. E. Cheatham, J. Šponer and M. Otyepka, *Journal of Chemical Theory and Computation*, 2010, **6**, 3836-3849.
17. A. Pérez, I. Marchán, D. Svozil, J. Sponer, T. E. Cheatham III, C. A. Laughton and M. Orozco, *Biophysical Journal*, 2007, **92**, 3817-3829.
18. W. L. Jorgensen, J. Chandrasekhar, J. D. Madura, R. W. Impey and M. L. Klein, *The Journal of Chemical Physics*, 1983, **79**, 926-935.
19. D. R. Roe and T. E. Cheatham, *Journal of Chemical Theory and Computation*, 2013, **9**, 3084-3095.
20. U. Essmann, L. Perera, M. L. Berkowitz, T. Darden, H. Lee and L. G. Pedersen, *The Journal of Chemical Physics*, 1995, **103**, 8577-8593.
21. J.-P. Ryckaert, G. Ciccotti and H. J. C. Berendsen, *Journal of Computational Physics*, 1977, **23**, 327-341.
22. D. A. Case, T. E. Cheatham, T. Darden, H. Gohlke, R. Luo, K. M. Merz, A. Onufriev, C. Simmerling, B. Wang and R. J. Woods, *Journal of computational chemistry*, 2005, **26**, 1668-1688.
23. Laboratory for Advanced Computing, University of Coimbra, http://www.lca.uc.pt
24. W. Humphrey, A. Dalke and K. Schulten, *Journal of molecular graphics*, 1996, **14**, 33-38.
25. G. D. Hawkins, C. J. Cramer and D. G. Truhlar, *Chemical Physics Letters*, 1995, **246**, 122-129.
26. J. Weiser, P. S. Shenkin and W. C. Still, *Journal of Computational Chemistry*, 1999, **20**, 217-230.
27. B. R. Miller, T. D. McGee, J. M. Swails, N. Homeyer, H. Gohlke and A. E. Roitberg, *Journal of Chemical Theory and Computation*, 2012, **8**, 3314-3321.
